# From the different pathogenesis of epileptogenesis: vitamins as an adjunctive treatment for epilepsy

**DOI:** 10.1186/s42494-025-00228-0

**Published:** 2025-07-09

**Authors:** Weiyi Sun, Yiming Wang, Bo Xiao, Zhaohui Luo

**Affiliations:** 1 Department of Neurology, Xiangya Hospital, Central South University, Changsha, 410008 China; 2 Xiangya School of Medicine, Central South University, Changsha, 410008 China; 3 National Clinical Research Center for Geriatric Disorders, Xiangya Hospital, Central South University, Changsha, 410008 China; 4 Clinical Research Center for Epileptic Disease of Hunan Province, Central South University, Changsha, Hunan 410008 China; 5 Department of Neurology, Xiangya Hospital, Central South University, (National Regional Center for Neurological Diseases), Nanchang, Jiangxi 330000 China

**Keywords:** Epilepsy, Vitamins, Antiseizure medications, Pro-inflammatory cytokines, ROS

## Abstract

Vitamins play an essential role in the maintenance of normal physiological functions of the human body. In recent years, the use of vitamins as an adjunctive treatment for epilepsy has attracted increasing interest academically. There is a substantial body of evidence indicating that vitamin supplementation could contribute to the treatment and prevention of epilepsy. This review  discusses the pathogenesis of epilepsy associated with ten vitamins from five perspectives, namely,  inflammatory signaling pathways, excitotoxicity, oxidative stress, neuroprotection, and the blood-brain barrier, and explores the relationships between the gut microbiota and vitamins in epileptic disorders with a focus on summarizing the antiepileptic effects of vitamin D and vitamin E. In addition, we discuss the effects of antiseizure medications on vitamins. This review aims to provide a more comprehensive view of the use of vitamins as an adjunctive therapy in epilepsy.

## Background

Epilepsy, a complex neurological disorder characterized by recurrent unprovoked seizures, affects more than 50 million individuals globally, with disproportionately high disease burdens in low- and middle-income countries with limited access to advanced therapies [1].

Seizure episodes are defined as "transient symptoms and/or signs due to abnormal and simultaneous neuronal activity of a population of neuronal cells in the brain" [2]. These episodes arise from diverse etiologies including acquired brain injuries (e.g. stroke and trauma), infections, autoimmune disorders, and genetic mutations, although approximately 30% of cases remain idiopathic [3]. Emerging evidence indicates that increases in excitatory neurotransmitters (e.g. glutamate [Glu]) and decreases in the inhibitory neurotransmitter γ-aminobutyric acid (GABA) contribute to the occurrence of seizures [4]. In addition, oxidative stress (OS) and the production of cytokines such as interleukin (IL)-1β and tumor necrosis factor-α (TNF-α) impact neuronal excitability and synchronicity [5]. This modulation of receptor function and expression contributes to the chronic nature of epilepsy [5].

While antiseizure medications (ASMs) remain the cornerstone of epilepsy management, 30–40% of patients develop drug-resistant epilepsy (DRE), and chronic ASMs use is associated with significant neuropsychiatric and systemic adverse effects, including cognitive impairment, osteoporosis, and teratogenicity [6]. These limitations underscore the urgent need for safe adjuvant therapies targeting the multifactorial pathophysiology of epilepsy. Compelling evidence suggests that micronutrient modulation, particularly vitamin supplementation, could offer a novel therapeutic strategy through pleiotropic neuroprotective mechanisms[5, 7, 8].

Since a wealth of studies have attested that neuroinflammation, glutamate excitotoxicity and OS are major events occurring during brain damage, vitamins, as indispensable cofactors in cellular homeostasis and neuromodulation, are increasingly recognized for their therapeutic potential in epilepsy. In addition to their classical roles in metabolism, vitamins exhibit pleiotropic antiepileptic properties through redox modulation, epigenetic regulation, and neuroimmune crosstalk [5, 9–12]. Emerging evidence positions vitamins A, B1, B6, B9, B12, C, D, E, K, and Q as potential modulators of epileptogenesis, with particular mechanistic insights documented for vitamin D (VD) and vitamin E (VE) [3, 13]. However, no study has comprehensively summarized the role of multivitamins in epilepsy and the possible regulation of related pathways.

**Fig. 1:**
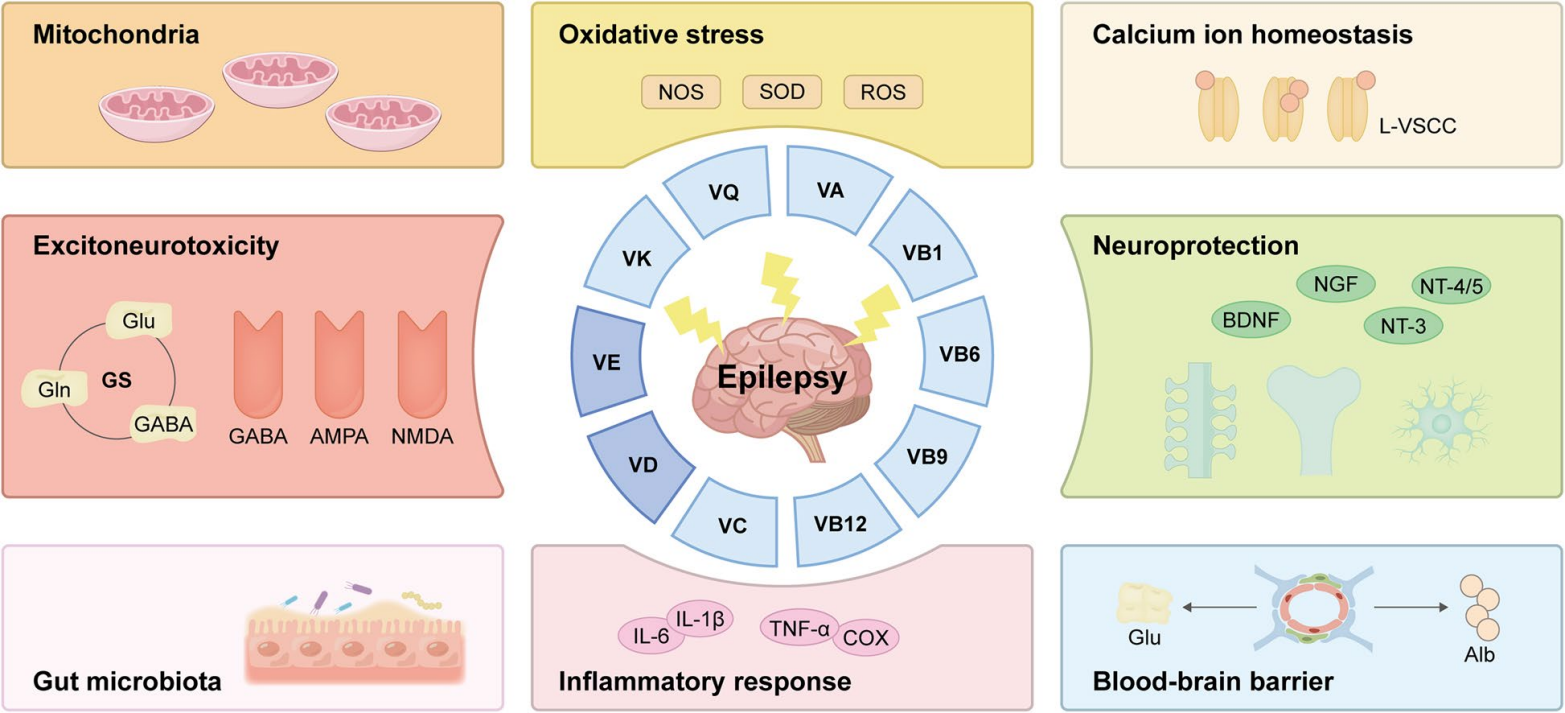
Schematic illustration of the antiepileptic mechanism of vitamins

This review synthesizes emerging experimental and clinical evidences to explore the multifaceted interplay between vitamins and epilepsy (Fig. 1). While epidemiological and preclinical studies increasingly suggest a potential modulatory role of vitamins in seizure pathophysiology, the bidirectional relationship, encompassing both the antiepileptic mechanisms of vitamins and the perturbing effects of epilepsy itself or ASMs on vitamin homeostasis, remains incompletely characterized. Our review offers a unique perspective for investigating supplementary treatments for epilepsy.

The roles of vitamins in different mechanisms of epileptogenesis include suppressing the inflammatory response (e.g. down-regulating TNF-α, IL-6, IL-1β and cyclooxygenase [COX] expression, etc.) and excitoneurotoxicity (e.g. down-regulating glutamine synthetase [GS] content, decreasing the re-uptake of Glu and inhibiting the breakdown of GABA, etc.), counteracting OS (e.g. down-regulating reactive oxygen species [ROS], superoxide dismutase [SOD], and nitric oxide synthase [NOS] levels, etc.), providing neuroprotective (e.g. up-regulating brain-derived neurotrophic factor [BDNF], nerve growth factor [NGF], neurotrophin [NT]-3 and NT-4/5 expression, etc.), attenuating blood-brain barrier (BBB) disruption, improving mitochondrial function, maintaining calcium ion homeostasis (e.g. down-regulating L-type voltage-sensitive Ca^2+^ channel [L-VSCC] expression), and regulating the gut microbiota.

## The roles of vitamins in different mechanisms of epileptogenesis

While traditional etiological frameworks emphasize genetic, structural, and synaptic remodeling mechanisms [14], recent preclinical evidences position vitamin-mediated pathways as potential countermeasures to several epileptogenic processes, including the reduction of pro-inflammatory cytokines and excitoneurotoxicity, antioxidant function, neuroprotection, protection of the BBB, and the effects of gut microbiota.

### Vitamins and pro-inflammatory cytokines

Neuroinflammation plays a central role in epileptogenesis, driving maladaptive plasticity through microglial activation, astrocyte reactivity, and dysregulated cytokine signaling [15]. Activated microglia undergo morphological changes, adopting an amoeboid-like phenotype with shortened processes and enlarged somata, and polarize into pro-inflammatory M1 or anti-inflammatory M2 subtypes [16]. M1 microglia release cytokines such as TNF-α, IL-1β and IL-6 and activate nuclear factor kappa-B (NF-κB) pathways [16]. Conversely, M2 microglia promote tissue repair via anti-inflammatory mediators such as IL-10 [17]. Earlier reports have established a connection between elevated levels of pro-inflammatory cytokines released by M1 microglia and an increased frequency and total duration of seizures [18]. Indeed, the wide array of pro-inflammatory cytokines (IL-1β, IL-6 and TNF-α) released during and after seizures activate inflammatory pathways in neurons and glial cells, and promote BBB leakage, which facilitates inflammatory cell infiltration [3]. This activation can change neuronal excitability and synchronization by modulating the function and expression of receptors, ultimately contributing to the development of epilepsy [5]. Clinical evidence has shown that elevated IL-1β levels in the peripheral blood of children with intractable temporal lobe epilepsy, correlating with onset time of a single convulsion [19]. Compared with controls, experimental models demonstrate mechanistic causality: the levels of microglial-derived IL-1β, IL-6 and TNF-α are rapidly elevated in the hippocampi of rodents with seizures [18]. These key seizure-induced pro-inflammatory cytokines are involved in various mechanisms of inflammatory pathways, which may contribute to the development of epilepsy in the process of inducing apoptosis, promoting the hyper-excitability, reducing the inhibitory drive, and regulating the re-uptake and release of neurotransmitters (Table 1).

**Table 1:** Mechanisms by which different pro-inflammatory cytokines induce seizures

Pro-inflammatory cytokines	Mechanism
IL-1β	Activating Src family and subsequent N-methyl-D-aspartate receptor (NMDA) receptor NR2 A/B subunit phosphorylation, which enhances NMDA receptor-mediated calcium influx [20]
	Promoting the hyper-excitability of neurons by decreasing the re-uptake of Glu in astrocytes and enhancing the release of Glu, thereby increasing the total concentration of extracellular Glu [21]
	The pathophysiological concentration of IL-1β in temporal lobe epilepsy (TLE) reduces GABA-mediated inhibitory neurotransmission by up to 30%, leading to neuronal hyperexcitability [22]
IL-6	Increased IL-6 could reduce hippocampal neurogenesis and is involved in oligodendrogliogenesis and astrogliogenesis [23]
	Exposure to IL-6 during pregnancy could cause changes in fetal hippocampal structure and morphology, increasing the risk of inflammatory neurodegeneration of the hippocampus with impaired spatial learning [24]
TNF-α	Inducing endocytosis of GABA_A_ receptors, reducing surface GABA_A_ receptors and inhibiting inhibitory neurotransmission [25]
	Stimulating and up-regulating α-amino-3-hydroxy-5-methyl-4isoxazole-propionic acid receptor (AMPA receptor), which enhances calcium influx and causes neurotoxicity [26]
	Up-regulating glutaminase and connexin 32 hemichannel in microglia to increase microglial Glu release, causing excitoneurotoxicity [27]

**Fig. 2:**
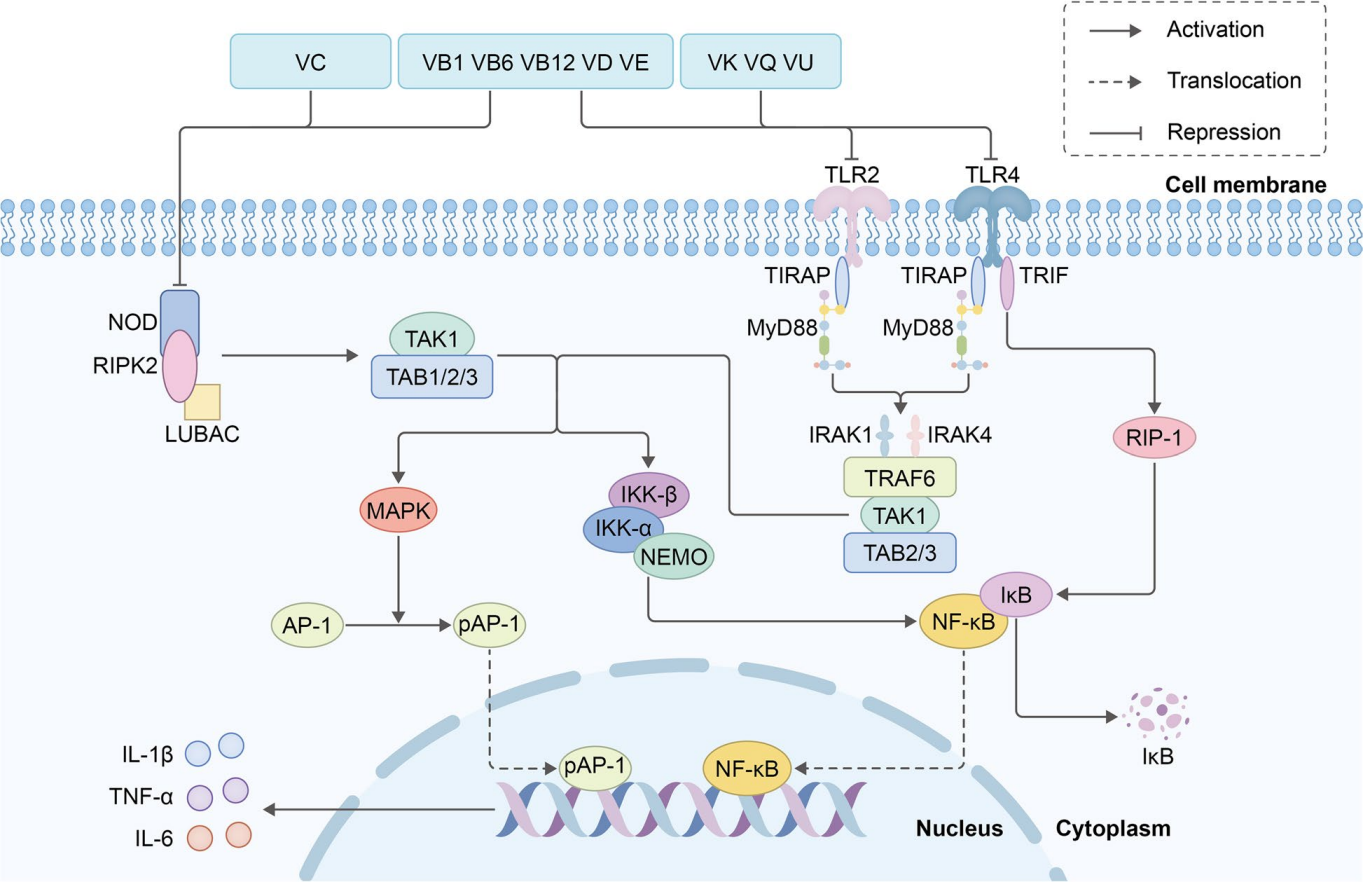
Schematic illustration of the vitamin-mediated reduction in pro-inflammatory cytokines through inhibiting TLR and NLR signaling pathways

The reduction in the production of pro-inflammatory cytokines by particular vitamins such as VD and VE is related mainly to Toll-like receptors (TLRs), which serve as key pattern recognition receptors (PRRs), acting as primary sensors [28]. Once recognizing the microbial components, TLRs immediately elicit innate immune responses and activate intracellular signaling pathways such as myeloid differentiation primary response gene 88 (MyD88), thereby activating the NF-κB or mitogen-activated protein kinase (MAPK) pathway, which culminates in the induction of pro-inflammatory cytokines such as TNF-α and IL-1β [28]. Like TLR, nucleotide-binding oligomerization domain-like receptors (NLRs) are cytosolic proteins that respond to various ligands such as bacterial and viral components [29]. The binding of NLR to ligands induces the activation of downstream NF-κB or MAPK pathways and subsequently leads to the secretion of pro-inflammatory cytokines [29]. These pathways contribute to recurrent seizures and epileptogenic network remodeling (Fig. 2).

Targeting these pathways represents a promising therapeutic strategy for epilepsy. VD exerts anti-inflammatory effects by suppressing TLR4/MyD88/NF-κB signaling and NLRP3 inflammasome assembly [30, 31]. Similarly, experimental studies has shown that treatment with coenzyme Q10 and VE, whether administered separately or together, results in blockade of the NF-κB/NLRP3 inflammasome pathway [32]. Interestingly, the combination outperforms a stronger anti-inflammatory effect, as it blocks the pathway more efficiently than single monotherapy (Table 2). Moreover, VE significantly attenuates hippocampal neuroinflammation by inhibiting astrogliosis and microglial reactivity and down-regulating microRNAs such as miR-146a, miR-124 and miR-126 [5].

**Table 2:** Vitamins modulating the TLR/NLR pathways in epilepsy-associated neuroinflammation

Vitamin	Mechanism of action	Experimental/clinical evidence
VD	Inhibiting TLR4/MyD88/NF-κB pathway-mediated neuroinflammation	Controlled cortical impact induced traumatic brain injury (rat model): ↓ hippocampal TNF-α, IL-1β and IL-6 [31]
	Suppressing NLRP3 inflammasome assembly by reducing the up-regulation of ROS	Surgery-induced post-operative cognitive dysfunction (mice model): ↓ hippocampal IL-18 and IL-1β [30]
VE	Reducing the expression of TLR2 and TLR4	Cisplatin-induced nephrotoxicity (rat model): ↓ renal tissues NF-κB, IL-6, and TNF [33]
	Suppressing NF-κB/NLRP3 inflammasome pathway	Heat stress-induced mood disturbances (mice model): ↓ prefrontal cortex NF-κB, caspase 1 and IL-1β [32]
VA	Inhibiting TLR4/MyD88/NF-κB pathway-mediated neuroinflammation via the activation of retinoic acid receptor α (RARα)	Surgery-induced post-operative cognitive dysfunction (mice model): ↓ hippocampal TNF-α, IL-1 [34]
VB complexes	Exhibiting high binding affinity to CD14 and TLR4/MD2, and subsequently suppressing LPS-induced pro-inflammatory cytokines	Lipopolysaccharides (LPS)-stimulated BV2 cells: ↓ cell supernatants TNF-α and IL-6 [35]
VB3	Silent mating type information regulation 2 homolog-1 (SIRT1)/poly ADP-ribose polymerase (PARP-1)/NLRP3 cascade modulation	LPS-induced depression (mice model): ↓hippocampal TNF-α, IL-1β, NF-κB and caspase 1 [36]
VC	Inhibiting the activation of NLRP3 inflammasome by reducing the level of cytosolic mtDNA and ROS	C57BL/6 mice, accelerated presbycusis model (mice model): ↓ cochlea, inferior colliculus and auditory cortex IL-1β, IL-18, IL-6 and TNF-α mRNA [37]
VK2 (MK-4)	Inhibiting NF-κB activation via the repression of IκB kinase (IKK)α/β phosphorylation	LPS-stimulated Human monocytic THP-1 and mouse RAW264.7 cells: ↓ cell supernatants NF-κB and IL-6 [38]
CoQ10	Suppressing NF-κB/NLRP3 inflammasome pathway	Heat stress-induced mood disturbances (mice model): ↓ prefrontal cortex NF-κB, caspase 1 and IL-1β [32]
	Suppressing TLR4/NF-κB signaling by reducing the ROS expression	C6-induced glioblastoma (rat model): ↓ brain tissue TNF-α and IL-1β [39]

The vitamin-mediated reduction in pro-inflammatory cytokine production is mainly associated with the TLR and NLR signaling pathways. In response to the ligands of these receptors, TLRs and NLRs immediately elicit innate immune responses and induce the activation of downstream NF-κB or MAPK pathways, which culminates in the induction of pro-inflammatory cytokines such as IL-1β, TNF-α, and IL-6. Among the vitamins examined, VK, VQ, VU, VD, VB1, VB6, VB12, and VE could inhibit the activation of TLR signaling pathway, and VD, VB1, VB6, VB12, VE, and VC could inhibit the activation of NLR signaling pathway.

### Vitamins and excitotoxicity

The dysregulation of glutamatergic-GABAergic equilibrium remains a pathophysiological cornerstone of epileptic hyperexcitability. Glu, the predominant excitatory neurotransmitter, induces neuronal hyperexcitability through its interaction with ionotropic receptors, such as AMPA, NMDA, and kainate, and metabotropic receptors, known as mGluRs [3]. Excessive NMDA receptor activation triggers Ca^2+^ influx, leading to mitochondrial dysfunction and OS, ultimately resulting in the death of neurons due to excitotoxicity [21]. AMPA receptor up-regulation further amplifies depolarization, whereas mGluR5 dysregulation in astrocytes perpetuates neuroinflammation and epileptiform activity [4]. On the other hand, pro-inflammatory cytokines (e.g. IL-1β and TNF-α) enhance NMDA-mediated glutamate release, forming a vicious cycle of hyperexcitability and inflammation [4].

Conversely, GABA, the principal inhibitory neurotransmitter, works to counteract excitation via GABA_A_ and GABA_B_ receptors. Genetic mutations in GABA_A_ subunits such as *GABRA1* or *GABRG2* disrupt chloride ion flux, reducing inhibitory postsynaptic currents [40]. Additionally, impaired GABA synthesis, due to glutamic acid decarboxylase (GAD1) dysfunction, diminishes synaptic GABA levels, further contributing to excitability [4]. Moreover, traumatic brain injury or genetic defects in GABA transporter (GAT) further hinder the reuptake of GABA, diminishing inhibitory tone [41]. This situation is often observed in pharmacoresistant epilepsy, where there is a loss of GABAergic interneurons or desensitization of the receptors, making ASMs ineffective.

**Fig. 3:**
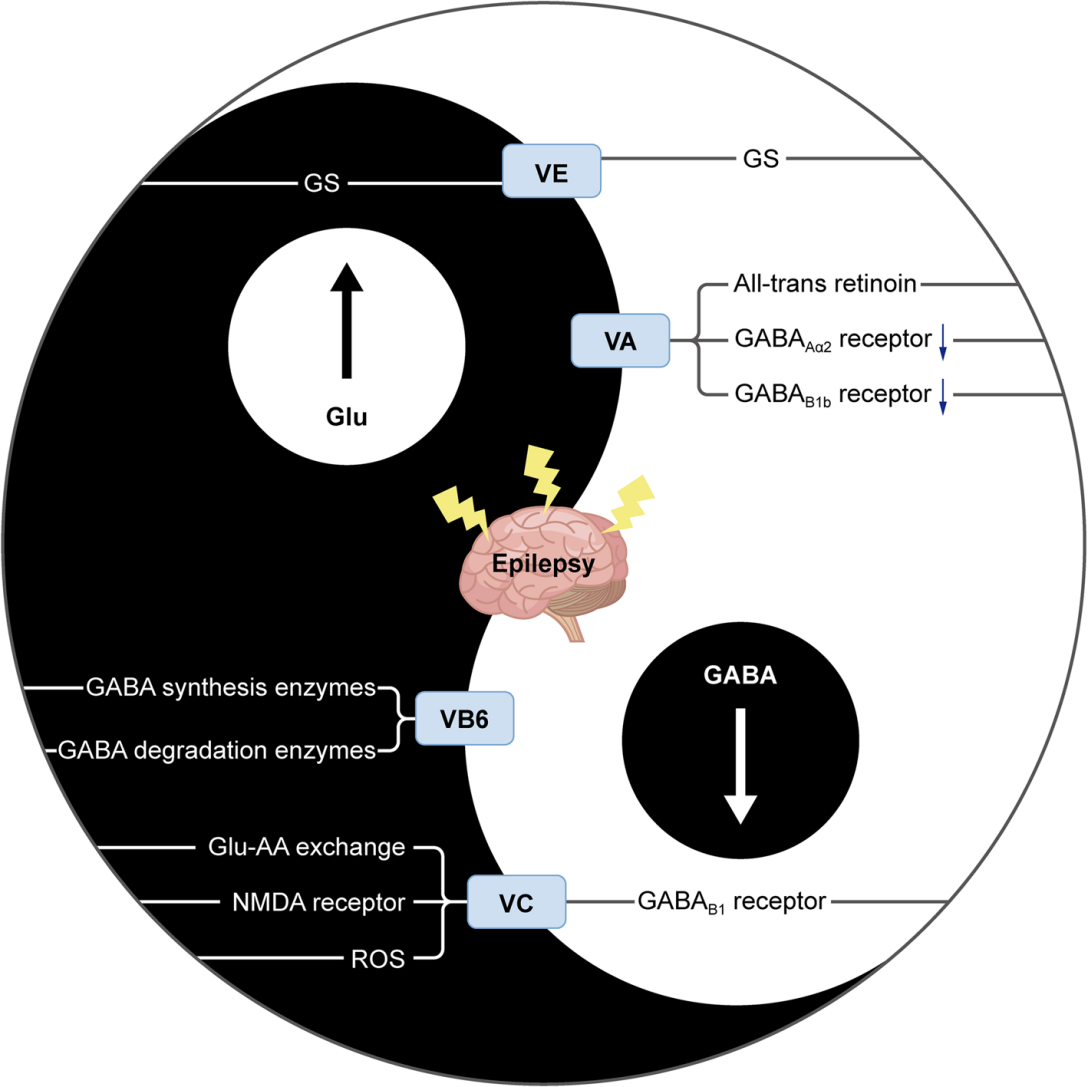
Schematic diagram illustrating the regulatory mechanisms involved in attenuating excitotoxicity through the actions of VA, VC,VB6 and VE

Altogether, the interplay between glutamatergic hyperactivity and GABAergic insufficiency underpins seizure initiation and propagation. VD has indirect neuromodulatory potential via the suppression of pro-inflammatory cytokines (IL-1β, TNF-α) and ROS—both of which are known to exacerbate glutamate release and impair astrocytic Glu reuptake [30]. Notably, GS, as a pivotal astrocytic enzyme, maintains glutamatergic-GABAergic equilibrium by converting synaptically released glutamate into glutamine, thereby preventing excitotoxicity and sustaining inhibitory GABA synthesis [42, 43]. In temporal lobe epilepsy (TLE), GS deficiency disrupts glutamate clearance, elevating extracellular glutamate and depleting glutamine, which impairs GABA production, exacerbating excitatory-inhibitory imbalance and seizure susceptibility [3, 43]. By targeting GS-mediated neurotransmitter homeostasis, pharmacological doses of VE could restore GS levels through scavenging the ROS generated during seizures and suppressing protein kinase Cδ (PKCδ), a negative regulator of GS expression [3]. In addition, GABA, which is recognized as the primary inhibitory neurotransmitter in the central nervous system, along with its receptors, is considered a key target for both current and future antiepileptic medications. Emerging studies have shown that VB6 could enhances GABA synthesis by up-regulating GAD, catalyzing glutamate-to-GABA conversion, and promoting vesicular GABA transport [44]. Similarly, the ability of VA to up-regulate the expression of GABA receptor expression (GABA_Aα2_/GABA_B1b_) has been demonstrated, which could be ascribed, at least in part, to gut microbiota regulation [9] (Table 3, Fig. 3).

**Table 3:** Vitamins modulating the glutamatergic-GABAergic equilibrium

Vitamin	Target pathway/receptor	Mechanism of action
VD	Pro-inflammatory cytokines (IL-1β, TNF-α) and ROS	Reducing IL-1β/TNF-α/ROS-induced glutamate release and astrocytic Glu reuptake impairment [30]
VE	GS	-Scavenging ROS to protect GS activity from oxidative degradation -Inhibiting PKCδ, a negative regulator of GS expression -Inhibiting microglial activation, indirectly stabilizing glutamate transporters and receptor dynamics in astrocytes [3]
	Mg^2+^	-Blocking NMDA receptors -Regulating Akt/mTOR pathway [45]
VB6	GAD and GABA transport	-Up-regulating GAD67 to catalyze glutamate-to-GABA conversion -Enhancing vesicular GABA transport [44]
VA	GABA_Aα2_/GABA_B1b_ receptors and gut-brain axis	-Up-regulating GABA_Aα2_/GABA_B1b_ receptor expression via retinoic acid-mediated signaling -Modulating gut microbiota (e.g. Lactobacillus) to up-regulate retinoic acid synthesis [9]
VC	NMDA receptors and ROS	-Inhibiting NMDA receptor activation -Reducing TNF-α/ROS-induced glutamate release and astrocytic Glu reuptake impairment [46]

One of the factors that contributes to the development of epilepsy is excitotoxicity. Epilepsy is associated with the down-regulation of GABA_Aα2_ and GABA_B1b_receptors due to a shortage in VA. VA undergoes metabolic processes in animals and leads to the formation of all-trans retinoic acid. This compound has the ability to direct the differentiation of embryonic stem cells into GABAergic neurons and has anticonvulsant effects. On the other hand, VC counteracts the reduction in GABA_B1_ receptors, diminishes the levels of ROS generated by Glu, facilitates the uptake of Glu through a mechanism involving Glu-ascorbic acid (AA) exchange, and inhibits the functioning of NMDA-gated channels. Additionally, VE plays a regulatory role in the Glutamine (Gln)-Glu-GABA cycle by maintaining an appropriate concentration of GS. Lastly, pyridoxine could affect GABA turnover in the mouse hippocampus by regulating GABA synthesis and degradation enzymes, affecting epilepsy from the perspective of neurotransmitters.

### Vitamins and ROS

The brain, as the most metabolically active organ in the human body, accounts for 20% of total oxygen consumption despite constituting only 2% of body mass [47]. This extraordinary energy demand renders neurons particularly vulnerable to OS due to their high mitochondrial density with intense electron transport chain activity, enrichment of redox-active transition metals, and the relatively low endogenous antioxidant capacity compared with those of peripheral tissues [48]. During epileptogenesis, aerobic metabolism generates ROS including superoxide (O₂⁻), hydrogen peroxide (H₂O₂), and nitric oxide (NO) through multiple pathways including mitochondrial electron leakage, nicotinamide adenine dinucleotide phosphate (NADPH) oxidase activation, and xanthine oxidase up-regulation. These radicals would ultimately disrupt neuronal integrity and synaptic plasticity [3].

NMDA receptor overactivation induces Ca^2+^ influx, stimulating NADPH oxidase (NADPH-OX)2 and mitochondrial electron transport chain leakage, which amplify O₂⁻ and H₂O₂ [3, 49]. These radicals subsequently initiate lipid peroxidation, which modifies ion channel kinetics (particularly voltage-gated sodium and potassium channels), while protein carbonylation alters GABA receptor trafficking-collectively promoting neuronal hyperexcitability [47]. Moreover, free radical generation can trigger seizure activity by directly inactivating GS, which leads to an abnormal accumulation of the excitatory neurotransmitter glutamic acid [50]. This process also results in a decrease in the brain's levels of GABA, the main inhibitory neurotransmitter [43]. Crucially, ROS act as second messengers in seizure-associated neuroinflammation by activating the NF-κB and activator protein-1 (AP-1) transcription factors, amplifying pro-inflammatory cytokine production such as IL-1β and TNF-α through TLR4/MyD88 signaling [51]. Concurrently, the up-regulation of pro-inflammatory cytokine further activates NADPH-OX and COX-2, resulting in the perpetuation of OS [3]. Notably, NO exhibits dual roles—physiological concentrations modulate cerebral blood flow via cyclic guanosine monophosphate (cGMP) pathways, whereas pathological levels inhibit cytochrome c oxidase, impair mitochondrial respiration, and induce glutamate excitotoxicity through NMDA receptor S-nitrosylation [52]. Altogether, these results suggest that ROS play a vital role in seizures and epileptogenesis.

**Fig. 4:**
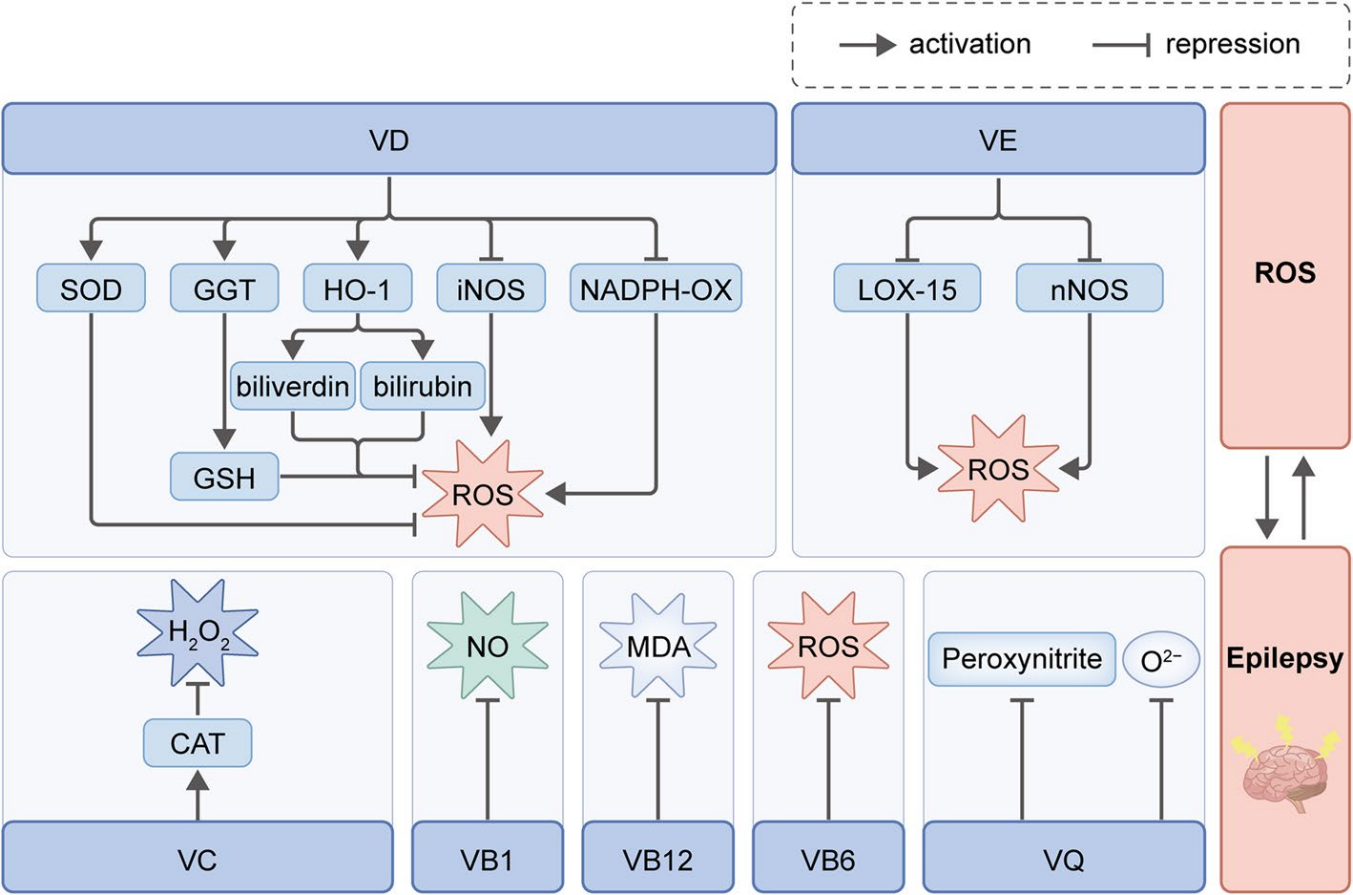
Schematic illustration of the vitamin-mediated regulation of ROS levels

Since emerging studies have underscored free radicals can act as pathogens in the epilepsy disease, natural compounds with antioxidant properties have been investigated for their potential role in preventing seizure-induced pathology. VD and VE, as natural antioxidants, have been proved to have beneficial effects in epilepsy, specifically in reducing convulsive behavior and alleviating brain OS. VD mitigates mitochondrial ROS via astrocytic glutathione (GSH) up-regulation and γ-glutamyl transpeptidase (GGT) activation [7], while VE preferentially quenches lipid peroxidation chains, potentiating endogenous antioxidants (SOD and catalase) in preclinical models [53]. In addition, a wealth of evidences have confirmed the protection effect of other vitamins including VC, VB1, VB6, VB12 and VQ, in counteracting OS [10, 54–57] (Table 4, Fig. 4).

**Table 4:** Mechanisms of vitamins in suppressing oxidative stress in epilepsy

Vitamin	Mechanism of Action	Experimental/Clinical Evidence
VD	Vitamin D receptor (VDR)-dependent inhibition of NADPH oxidase activity	Non-obese type 2 diabetes model(rat model): ↓ aorta NADPH p22 phox, NADPH p47 phox [58]
	Down-regulating NO expression through inhibiting inducible nitric oxide synthase (iNOS) in activated microglia	Carrageenan-induced paw edema(mice model): ↓ hippocampus and prefrontal cortex iNOS and COX-2 [59]
	Maintaining GSH pools via up-regulating glutamate cysteine ligase (GCLC) and glutathione reductase (GR)	High-glucose exposed U937 monocytes:: ↑ expression of GCLC and GR, levels of GCLC protein and GR activity, and formation of GSH [60]
	Enhancing heme oxygenase-1 (HO-1) expression via SIRT1-mediated NRF2 deacetylation, thus facilitating heme degradation and bilirubin production	D-Galactose induced Alzheimer disease: ↑ brain homogenates NRF2 and HO-1 [61]
VE	Scavenging free radicals and inhibits lipid peroxidation via the redox recycling and H atom donating ability	Pilocarpine-induced seizures (mice model): ↓ hippocampal lipid peroxidation level and nitrite content; ↑ hippocampal superoxide dismutase and catalase [53]
VB1	Preventing NO/cGMP signaling	Pentylenetetrazole-induced seizures(mice model): ↓ hippocampal caspase-3, NO, and cGMP levels [54]
VB12	Reducing pro-inflammatory cytokines-induced oxidative stress	Clinical trials (chronic pancreatitis): ↑ GSH, glutathioneperoxidase (GPx) and SOD [62]
VC	Directly neutralizing ROS and decreasing lipid peroxidation	Clinical trials (temporal lobe epilepsy): ↓ H_2_O_2_ [63]
CoQ10	Scavenging free radicals through interconversion between three redox states	Kainate induced TLE (rat model): hippocampal malondialdehyde (MDA) and nitrite content [64]

VD could promote ROS scavenging through enhancing the enzymatic activity of SOD, promoting the up-regulation of GGT expression and stimulating the HO-1 signaling pathway. SOD functions as an antioxidant by effectively scavenging ROS. GGT could enhance the concentration of GSH, a crucial endogenous antioxidant. HO-1 promotes the synthesis of powerful endogenous antioxidants biliverdin and bilirubin. SOD, GSH, biliverdin, and bilirubin possess significant antioxidant effects. Moreover, VD has the ability to counteract the action of NADPH-OX and inhibit the expression of iNOS, thereby reducing the generation of ROS. VE significant reduced the expression of neuronal-type nitric oxide synthase (nNOS) and lipoxygenase 15 (LOX-15), thereby effectively reducing ROS production. VC has the potential to increase the synthesis of catalase (CAT), leading to the promotion of H_2_O_2_ scavenging. VB1 effectively reduces the concentration of NO and maintains NO expression at a precise level. Additionally, VB6 could decrease ROS levels to reduce OS. VB12 supplementation was found to preserve the appropriate levels of NO and malondialdehyde (MDA). VQ possesses powerful antioxidant properties, enabling it to effectively suppress the generation of ROS.

### Vitamins and neuroprotection

**Fig. 5:**
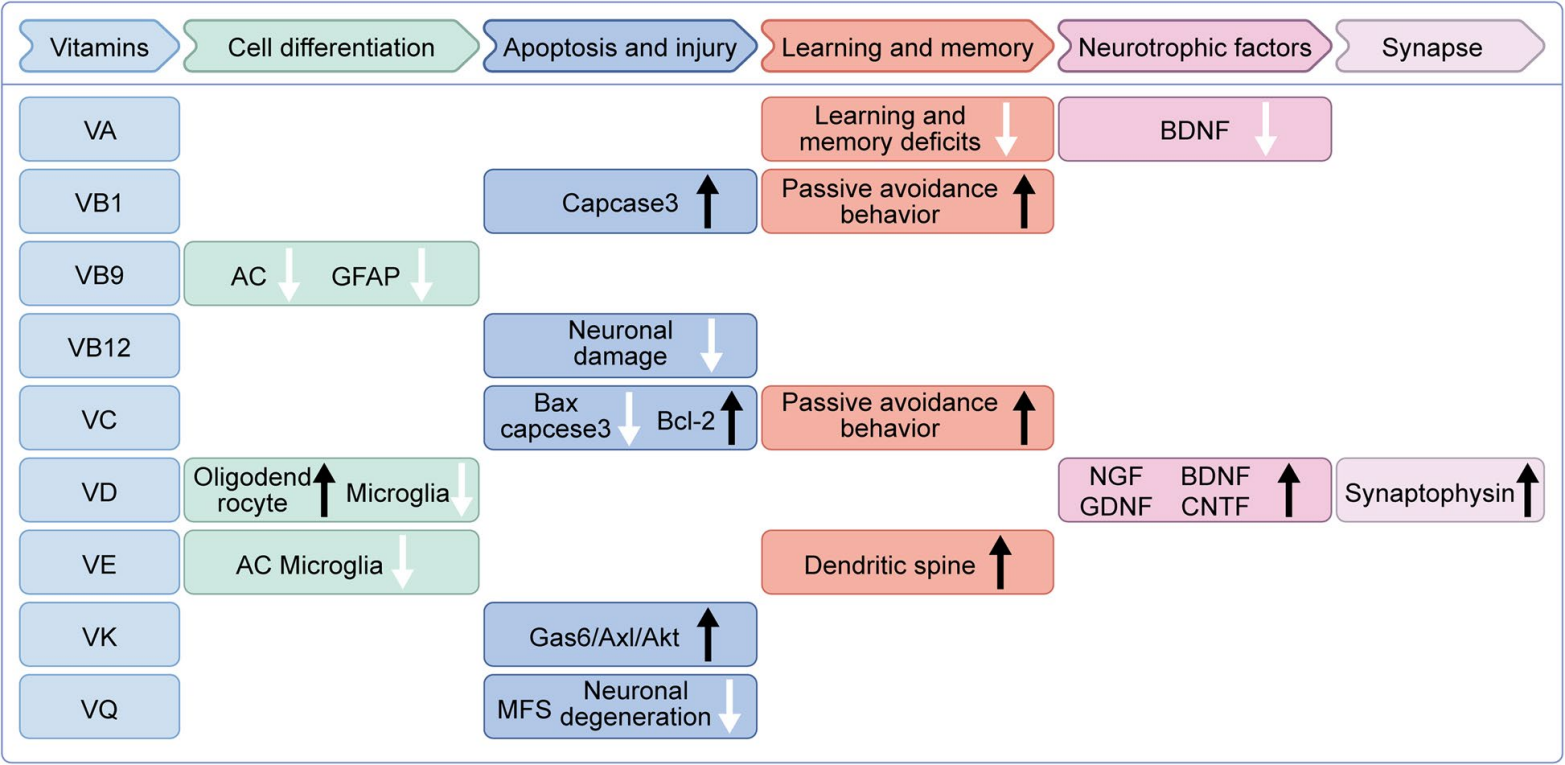
Schematic depiction of the potential efficacy of nine vitamins in mitigating the effects of epilepsy via neuroprotective mechanisms

Neurotrophins, a phylogenetically conserved family of secretory proteins comprising BDNF, NGF, neurotrophin-3 (NT-3), and neurotrophin-4/5 (NT-4/5), orchestrate multifaceted roles in neurodevelopment, synaptic plasticity, and neuronal survival across both the central and peripheral nervous systems [65]. Emerging preclinical evidence posits these molecules as potential modulators of neurodegenerative diseases, particularly through their capacity to mitigate neuroaxonal injury and maladaptive circuit reorganization [66]. VD supplementation has been found out to increase NT-3/NGF expression via genomic VDR signaling, potentially mitigating seizure-induced dendritic atrophy [67]. Concurrently, CoQ10 pretreatment could ameliorate hippocampal neuronal loss and aberrant mossy fiber sprouting in kainate model of TLE in rats [64] (Fig. 5).

The mechanisms underlying the neuroprotective effects of vitamins in the context of epilepsy could be classified into five aspects: neuronal cell differentiation, apoptosis and injury, learning and memory capacity, neurotrophic factors, and synapses.

Among the various vitamins, VA deficiency could down-regulate the expression of BDNF and aggravate the behavioral learning and memory deficits. VB1 improves passive avoidance performance, which indicates that it improves learning and memory ability. VB1 also could prevent apoptosis by reducing caspase-3. VB9 has been found to decrease the intensity of glial fiber acidic protein (GFAP) and inhibit the proliferation of reactive astrocytes. VB12 is able to improve the performance of rats in passive avoidance assays and reduce hippocampal neuronal damage. VC has been shown to reduce neuronal death by down-regulating the activation of Bcl2-associated X (Bax) and capcase3 while simultaneously increasing the expression of B-cell lymphoma-2 (Bcl-2). VD has been shown to up-regulate the expression of neurotrophic factors such as NGF, BDNF, ciliary neurotrophic factor (CNTF), and glial cell-derived neurotrophic factor (GDNF). Additionally, VD promotes the proliferation of neural stem cells and their differentiation into neurons and oligodendrocytes, while reducing astrocyte proliferation. VE has been shown to decrease the activation of astrocytes and microglia, reduce the expression of neuroglial proteins (e.g. GFAP), mitigate neuronal degeneration and cell death, and promote the production of dendritic spines. Furthermore, VE increases the optical density of synaptophysin immunoreactivity, indicating an enhancement in synaptic connectivity. The protective effect of VK on apoptosis is mediated through preservation of the Gas6/Axl/Akt pathway. Additionally, VQ significantly reduces moss fiber germination (MFS) intensity and neuronal degeneration in the CA1 and CA3 regions of the hippocampus.

### Vitamins and the BBB

The BBB acts as a critical interface between the systemic circulation and the central nervous system, maintaining the unique environment required for proper neural function. This specialized structure is primarily composed of endothelial cells (ECs) of blood vessels, which are tightly connected through paracellular tight junctions (TJs) and adherens junctions (AJs) that limit the uncontrolled passage of substances [68]. Clinical data and animal experiments suggest that BBB dysfunction such as impairments to TJs or AJs as well as changes in EC function, plays a critical role in the pathogenesis of seizures and epilepsy [69, 70]. BBB disruption facilitates epileptogenesis via multiple pathways: extravasation of neurotoxic substances (e.g. iron, thrombin, glutamate) directly induces neuronal hyperexcitability and network reorganization [71, 72], while albumin (Alb) leakage triggers TGFβ-mediated astrocytic activation, impairing potassium buffering and promoting excitatory synaptogenesis [73, 74]. Neuroinflammation, driven by peripheral immune cell infiltration and IL-1β signaling, further exacerbates epileptogenic processes [75]. However, not all BBB disruption leads to epilepsy, with outcomes influenced by factors such as lesion location, duration of dysfunction, and individual genetic/epigenetic predispositions [68].

While BBB dysfunction can contribute to epileptogenesis, seizures themselves can also disrupt BBB integrity. Seizures perpetuate BBB dysfunction through glutamate-induced matrix metalloproteinase (MMP) activation, oxidative stress, and neurovascular uncoupling [68].

**Fig. 6:**
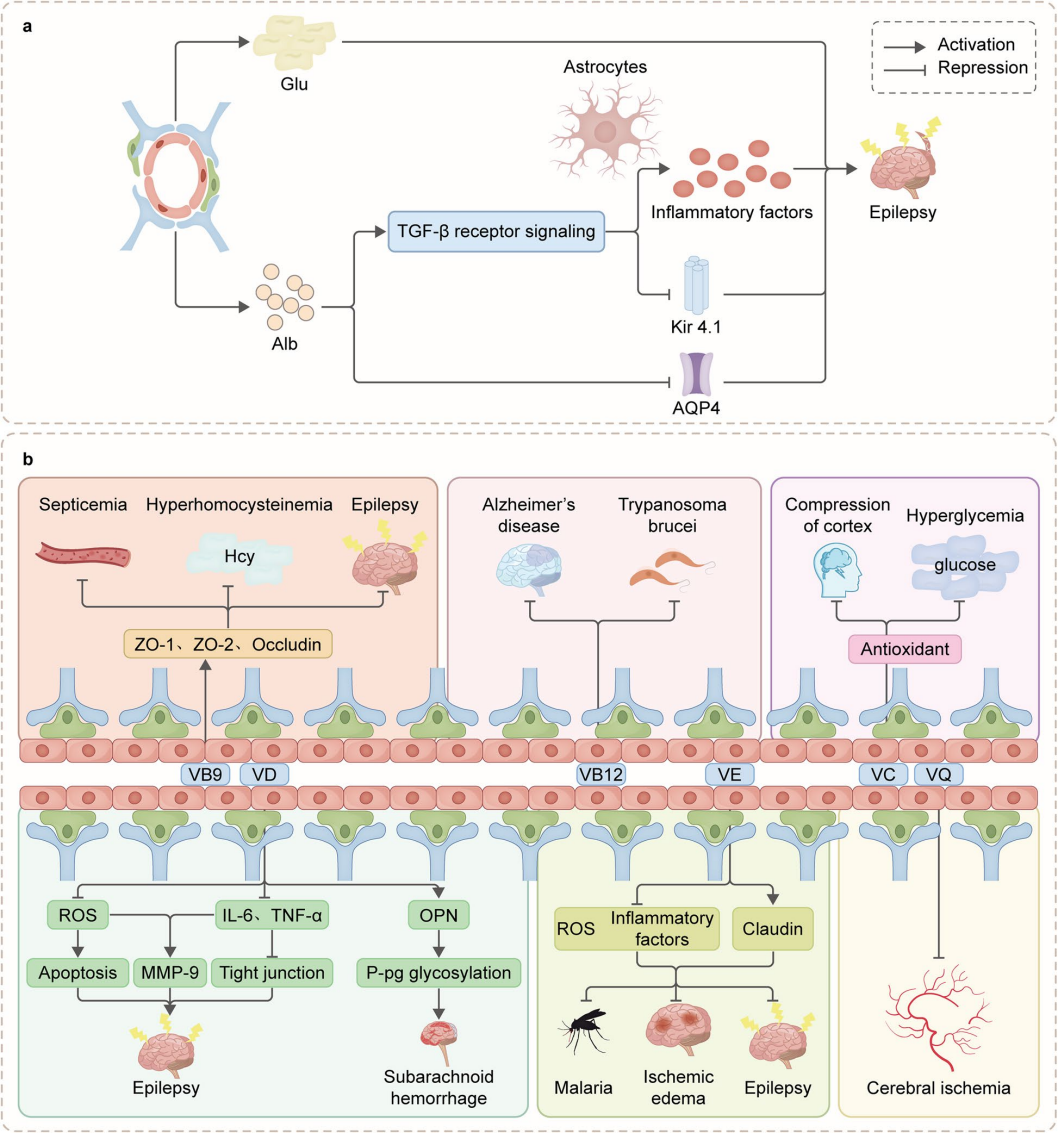
**a **Schematic picture of how the damage to BBB can worsen the condition of epilepsy. Dysfunction of the BBB leads to the translocation of serum constituents, including albumin and Glu, over the BBB and their subsequent access to neuronal cells. Astrocytes facilitate the internalization of Alb via TGF-βR, resulting in the suppression of inwardly rectifying potassium channel 4.1 (Kir4.1) gene expression and the stimulation of inflammatory chemical production. Exposure of the brain to albumin results in a comparable down-regulation of water channel protein-4 (AQP4) expression. All of the above processes exacerbate the epileptic process. **b **The potential mechanisms underlying the antiepileptic effects of these six vitamins involve their ability to maintain the integrity of the blood-brain barrier

Extensive research has demonstrated the involvement of BBB dysfunction in epileptogenesis, suggesting that targeting BBB repair could offer a valuable therapeutic approach for preventing epilepsy in these patients. In addition to counteracting BBB hyperpermeability by inhibiting the production of pro-inflammatory cytokines and ROS, VD has been shown to exert direct effects on ECs by up-regulating the expression of TJ proteins such as zonula occluden (ZO)-1 and claudin-5 while simultaneously down-regulating the levels of cell adhesion molecules such as ICAM-1 and VCAM-1, which collectively promotes the stabilization of the BBB [76]. Similarly, the ability of VE to reduce neuroinflammation and up-regulate claudin expression would promote BBB recovery [5]. Moreover, NA-4OCH3, a nicotinamide derivate, has been demonstrated to increase the integrity of both human and rat cell-based BBB models via elevating claudin-5 level and restoring the activity of glutathione, and the mitochondrial membrane potential [77] (Fig. 6).

VB9 has been shown to attenuate BBB disruption in conditions such as sepsis, hyperhomocysteinemia, and epilepsy. This effect is believed to occur through a biochemical process involving the restoration of tight junction proteins, including ZO-1, ZO-2, and occludin. VB12 has been shown to provide protection to the BBB affected by Alzheimer's disease and trypanosomiasis. VC provides protection to the BBB under conditions of sustained pressure in the somatosensory cortex and hyperglycemia. VD could reduce apoptosis by inhibiting ROS, increase expression of tight junction proteins by inhibiting pro-inflammatory cytokine production, and protect the BBB by inhibiting MMP-9 expression through the inhibition of ROS and pro-inflammatory cytokine production. In subarachnoid hemorrhage, VD increases the expression of osteopontin (OPN) to promote the glycosylation of P glycoprotein (P-gp), which protects the BBB by effectively removing foreign substances from the cells. On the other hand, VE protects the BBB in conditions such as malaria, ischemic edema, and epilepsy. The underlying molecular mechanism behind this protective effect is associated with the reversal of claudin down-regulation, a protein crucial for maintaining the integrity of the BBB. VQ could protects the BBB during cerebral ischemia.

### Vitamins and the gut microbiota

There is a complex and metabolically active microbial ecosystem in the human gut, which is essential to human systemic metabolism and health [78]. The gut-microbiota-brain axis (GMBA), as the bidirectional communication network between the central nervous system and the enteric nervous system, emerges as a dynamic interface in epilepsy pathophysiology [79]. Numerous clinical and preclinical studies suggest that gut dysbiosis may contribute to epilepsy development [80, 81]. Medel-Matus et al. reported that fecal microbiota transplantation (FMT) from stressed to sham-stressed rats accelerated kindling epileptogenesis, whereas the pro-epileptic effect of stress was counteracted in stressed rats treated with FMT from sham-stressed rats [82].

A recent study suggested that the relative abundances of the gut microbiota (e.g. Bacteroides, Cronobacter, Prevotella, and Bifidobacterium) were dramatically different between healthy individuals and patients [83]. Given the objective of this review, we posit that the administration of vitamin supplements to regulate the gut microbiota holds promise as a prospective therapeutic alternative for epilepsy. By enhancing microbial production of short-chain fatty acids (SCFAs), VD fosters extrathymic Treg differentiation and attenuates proinflammatory cytokine release in macrophages [84]. In addition to stimulating the production of SCFAs, VB12 supplementation exerts a protective effect on the intestinal epithelium [85]. What’s more, VC, VB2, VB2 + VC, VD and VE treatments increased the relative abundance of Bifidobacterium and the level of Actinobacteria [86]. Paradoxically, with VD treatment, there was a trend towards a decrease in Bacteroidetes, which is strongly associated with the seizure severity of epileptic patients [83, 86]. However, there is a lack of research investigating the relationships among vitamins, the gut microbiome, and epilepsy. Among the existing studies, the exploration of the modulatory effect of vitamins on the gut microbiota is limited concerning sample size, sex differences, habitual diet differences, and difficulty in accurately measuring the intake of vitamins. These limitations necessitate further investigation through studies with larger-scale designs and multi-omics approaches. It is premature to draw definitive conclusions at this juncture.

## Antiepileptic effects of vitamins

Building upon the pathophysiological frameworks outlined earlier, this section critically evaluates vitamins particularly VD and VE as multimodal therapeutic agents in epilepsy management to provide a direction for future research on vitamin therapeutic and prophylactic measures against epilepsy.

### Antiepileptic effects of VD

**Fig. 7:**
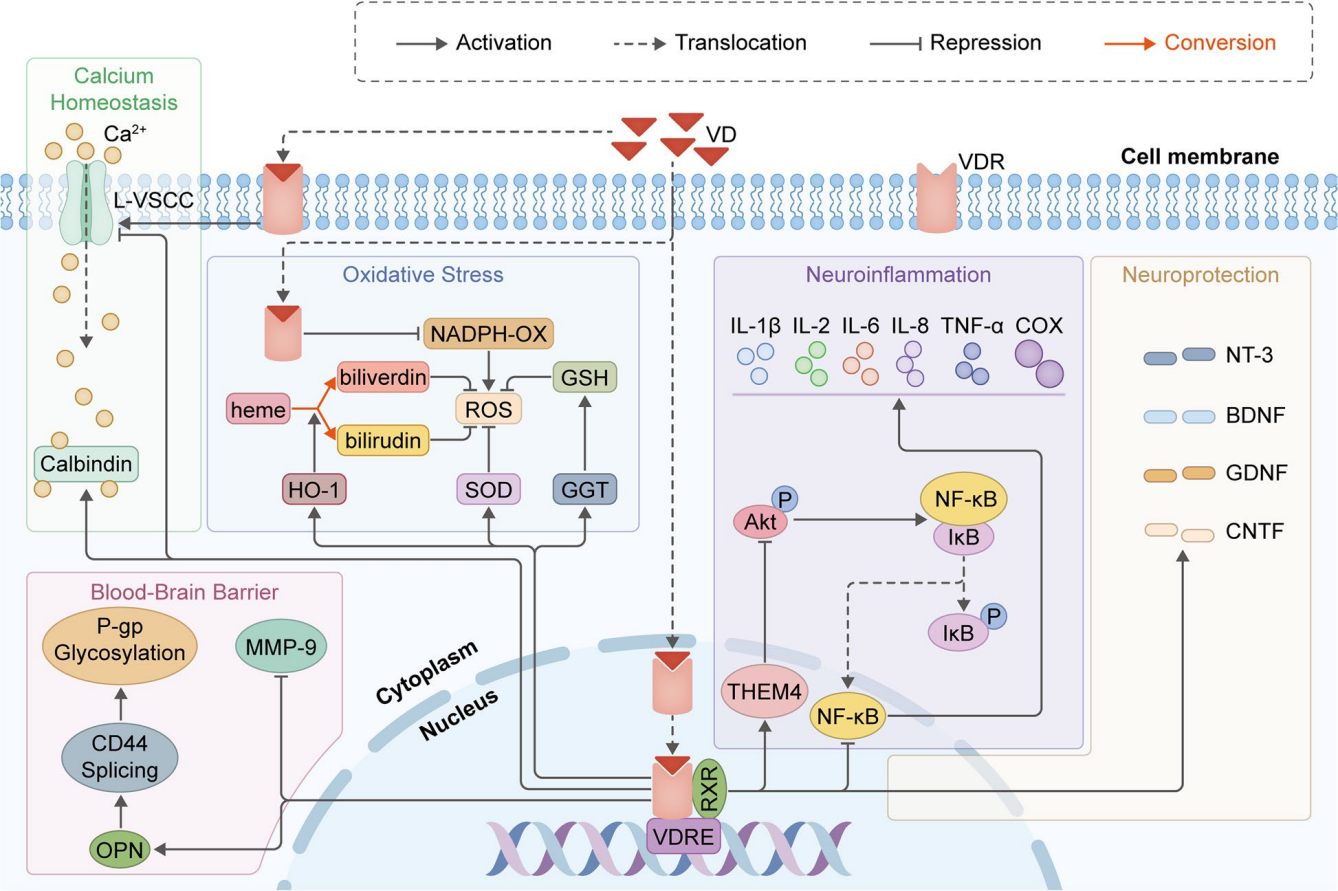
Schematic illustration of the VD antiepileptic mechanism

VD is a vital nutrient that plays a crucial role in various physiological processes in the human body. Its functions extend beyond the traditional understanding of bone health to encompass a wide array of biological activities, including immune modulation and neurological health. Increasing evidences have indicated that VD as a pleiotropic modulator has considerable potential in improving epilepsy management via VDR-mediated classical genomic and faster non-genomic functions [87]. For instance, daily supplement of 5000 IU VD3 supplementation dramatically increases the seizure threshold and reduces median seizure frequency in drug-resistant epilepsy patients [88]. As previously reported, the occurrence and development of epilepsy could be due to a variety of factors including genetic predisposition, developmental dysfunction and nerve injury [14]. Taking into account the existing research on the mechanism of action of VD in epilepsy, we present a concise overview of the following antiepileptic mechanisms associated with VD: down-regulation of pro-inflammatory cytokines expression, inhibition of the neuroinflammatory, regulation of calcium channels and calcium-binding proteins (CBP), up-regulation of neurotrophins expression, attenuation of BBB disruption, protection of the intestinal mucosal barrier, and modulation of the gut microbiota (Fig. 7).

The main antiepileptic mechanisms of VD include decreasing neuroinflammation, counteracting OS, providing neuroprotection, maintaining calcium homeostasis and attenuating BBB disruption. Regarding the neuroinflammation, VD induces the transcription of thioredoxin superfamily member 4 (THEM4) and subsequently inhibits Akt phosphorylation and the NF-κB pathway, ultimately resulting in the down-regulation of IL-1β, IL-2, IL-6, IL-8, TNF-α and COX expression. With respect to OS, VD could enhance the activity of antioxidant enzymes such as SOD and increase the expression of GGT which enhances the concentration of GSH and contributes to the promotion of ROS scavenging. VD also could stimulate the HO-1 signaling pathway, thus metabolizing free heme into the biliverdin and bilirubin, which are effective endogenous antioxidants. In addition, VD inhibits ROS production through antagonizing NADPH-OX activity. Regarding the neuroprotection, VD, acting as an inducer of neurotrophins, contributes to increase in NT-3, BDNF, GDNF and CNTF expression. VD attenuates BBB disruption through up-regulating the neuroprotective glycoprotein OPN expression, which in turn activates the CD44 splicing and P-gp glycosylation. Besides, VD treatment down-regulates MMP-9 expression, which could restore BBB permeability via improving TJ protein. Regarding the calcium homeostasis, lower concentrations of vitamin D hormone (VDH) selectively down-regulate L-VSCC expression and result in the neuroprotective effects, while higher concentrations of VDH lack neuroprotective action, accompanied by increased L-VSCC current. In addition, a cell culture study revealed that VD induces the expression of CB, which plays a critical role in regulating calcium homeostasis. 

#### VD exerts antiepileptic effects by down-regulating pro-inflammatory cytokines

Upon ligand activation the VDR could activate or repress gene transcription via genomic signaling pathways [89]. It has been reported that the 1,25(OH)2D3-VDR complex repressed the TLR4/MyD88/NF-κB and THEM4/Akt/NF-κB signaling pathways. In the hippocampal tissue of rats, 1,25(OH)2D3 reduces the level of LPS-induced TLR4, MyD88, and nuclear NF-κB p65 protein [31]. In addition, VD administration could activate the endogenous target factor silent mating type information regulation 2 homolog-1 (SIRT1), reducing transcriptional activity, and then inhibit NF-κB pathway-mediated neuroinflammation, as well as down-regulating the expression of the downstream signaling molecules TNF-α and IL-1β [61]. VD is neuroprotective against status epilepticus (SE)-mediated neuronal death.

In addition, VD exerts anti-neuroinflammatory effects by modulating microglial polarization. This modulation shifts the balance from pro-inflammatory M1 to anti-inflammatory M2 phenotypes [90], which is characterized by up-regulated anti-inflammatory cytokines (IL-4, IL-10 and TGF-β), neurotrophic factors, and enzymes like arginase 1 and HO-1, which enhance the ROS detoxification and the phagocytic clearance of neurotoxic debris, including myelin fragments [91].

#### VD exerts antiepileptic effects by inhibiting OS

VD demonstrates multimodal antioxidant properties through genomic and non-genomic mechanisms. The VDR, which is widely expressed in hippocampal neurons and astrocytes, mediates the transcriptional regulation of antioxidant genes containing VD response elements (VDREs). Substantial clinical data and animal experiments have demonstrated the potential benefits of VD in preventing seizures and epileptogenesis effects of OS [92].

The antioxidant effect of VD could be examined from two perspectives: inhibition of ROS production and promotion of ROS scavenging. The overproduction of NADPH oxidases is the major cause of excess ROS production in the injured brain microenvironment [3]. 800 IU/kg VD reduces ROS production via VDR-dependent inhibition of NADPH oxidase activity [58]. Furthermore, VD inhibits LPS-induced iNOS overexpression through both genomic (VDRE competition with NF-κB binding) and rapid non-genomic pathways, thus maintaining NO expression at a specific level and inhibiting NO-mediated neuronal damage and death [93]. On the other hand, ROS scavenging is associated with the body's antioxidant system, including SOD, CAT and GSH [93]. VD significantly up-regulates glutamate cysteine ligases and glutathione reductase to maintain reduced GSH pools [60]. Meanwhile, through SIRT1-mediated NRF2 deacetylation, VD enhances HO-1 expression, facilitating heme degradation and bilirubin production [61]. In addition, VD increases catalase mRNA stability through HuR protein interactions while enhancing the activity of antioxidant enzymes such as SOD to improve antioxidant capacity [94]. Notably, VD's antioxidant efficacy exhibited U-shaped dose dependency. Compared with insufficiency or sufficiency VD deficiency groups, severe VD deficiency significantly elevated systemic immune-inflammatory index levels [95]. Altogether, these findings suggest that VD, with its direct and indirect antioxidant effects, hold great potential for decreasing the severity of seizures and their detrimental effects on brain tissue and functions.

#### VD exerts antiepileptic effects by maintaining calcium homeostasis

VD exerts precise regulation across tiers of calcium signaling. Firstly, the L-VSCC as an important target of VD regulation of Ca^2+^ homeostasis demonstrates seizure-associated hyperactivity [96]. Brewer et al. conducted pivotal electrophysiological investigations revealing a concentration-dependent biphasic response of VDH in modulating L-VSCC dynamics and neuroprotection within hippocampal neuronal networks [97]. Their patch-clamp analyses demonstrated that physiologically relevant VDH concentrations (1–100 nM) induced selective transcriptional down-regulation of L-VSCC α1 subunits (Cav1.2/Cav1.3), which was correlated with reduced calcium influx and enhanced neuronal survival in glutamate-challenged cultures. Notably, this neuroprotective profile was abolished at supraphysiological concentrations (500–1000 nM), where paradoxical potentiation of L-VSCC currents coincided with loss of cytoprotective effects. In addition to its modulation of voltage-gated calcium channels, emerging evidence suggests that VD signaling intersects with CBP dynamics in maintaining neuronal calcium homeostasis. CBPs, particularly calmodulin (CaM), parvalbumin (PV), calretinin (CR), and calbindin-D28 K (CB), exert neuroprotective effects through dual mechanisms: rapid cytoplasmic Ca^2+^ buffering via high-affinity EF-hand domains and long-term transcriptional regulation of calcium extrusion pumps [98]. It has been reported that the administration of VD up-regulates CBP expression in the brain through VDR-mediated promoter activation, resulting in improvements in memory and cognition [99].

#### VD exerts antiepileptic effects by up-regulating neurotrophins expression

Intriguingly, VD metabolites appear to exert pleiotropic effects on neurotrophin dynamics, as demonstrated by in vitro and in vivo seizure models. A rodent study employing C57BL/6 female adult mouse neural stem cells (NSCs) revealed that chronic 1,25-(OH)2-D3 supplementation up-regulated the expression of NT-3, BDNF, GDNF and CNTF [100]. Research in rodent models has consistently demonstrated that supplement with 25(OH)D3 leads to increased expression of NGF and BDNF in central nervous system tissues, highlighting the potential neurotrophic effects of VD metabolites [101].

Furthermore, while VD enhances neurotrophin expression, emerging evidence cautions against oversimplification of this relationship. For instance, BDNF up-regulation in chronic epilepsy models exhibits dualistic properties, potentially exacerbating hyperexcitability through TrkB receptor-mediated potentiation of glutamatergic synapses—a phenomenon not yet systematically evaluated in VD intervention studies [102].

#### VD exerts antiepileptic effects by attenuating BBB disruption

The multimodal BBB stabilization mechanisms of VD warrant particular scrutiny given its neuroimmunological properties. Indeed, BBB disruption is closely associated with the redox homeostasis and inflammatory modulation [3]. Thus, the ability of VD to down-regulate the expression of ROS and pro-inflammatory cytokines could attenuate BBB disruption, maintaining cerebral homeostasis and suppressing epileptogenesis. In addition, VD up-regulates the expression of claudin-5/ZO-1 tight junction proteins through OPN-mediated Akt/p42/p44/MAPK phosphorylation, FAK/PI3 K/Akt and CD44/P-gp glycosylation pathways, which has protective effects against BBB dysfunction and apoptotic neuronal cell death [103–105]. Recent studies by de Oliveira and colleagues have further confirmed these observations, demonstrating that when mice with experimental autoimmune encephalomyelitis (EAE) receive 1,25(OH)2D3 supplementation, it not only enhanced the expression of ZO-1 but also effectively reduces BBB permeability, which is accompanied by noticeable alleviation of disease symptoms. Futhermore, 1,25(OH)2D3 has been demonstrated to reduce the expression of matrix metalloproteinase-9 (MMP-9), which plays a crucial role in degrading extracellular matrix components, including collagen, fibronectin, and laminin, as well as tight junction proteins, leading to BBB instability [106].

#### VD exerts antiepileptic effects by modulating the gut microbiota

Emerging evidence suggests that VD suppresses the NF-kB p65-mediated MLCK-P-MLC signaling pathway via direct VDR-transforming growth factor-β-activated kinase 1 (TAK1) interactions, thereby ameliorating the altered localization of TJ proteins and preserving tight junction continuity in colonic organoids [107]. The therapeutic potential of VD-mediated GMBA modulation requires cautious interpretation, due to lack of VD status monitoring in donor/recipient animals and the absence of microbial metabolite profiling.

### Antiepileptic effects of VE

VE refers to a group of eight fat-soluble compounds—specifically α-, β-, γ-, and δ-tocopherols, along with their corresponding α-, β-, γ-, and δ-tocotrienol counterparts—that are naturally synthesized in plants [108]. These compounds are found in varying concentrations in foods rich in fats, such as vegetable oils, nuts, and seeds [109]. Additionally, they are often added to fortified foods, where α-tocopherol (α-TOH) is typically the primary form used. α-TOH is the most biologically active form and is preferentially retained in human tissues due to its high affinity for the α-tocopherol transfer protein [110]. Moreover, tocotrienols have been shown to exhibit superior antioxidant properties compared with tocopherols, potentially due to their ability to penetrate cell membranes more effectively and modulate various signaling pathways [111].

**Fig. 8:**
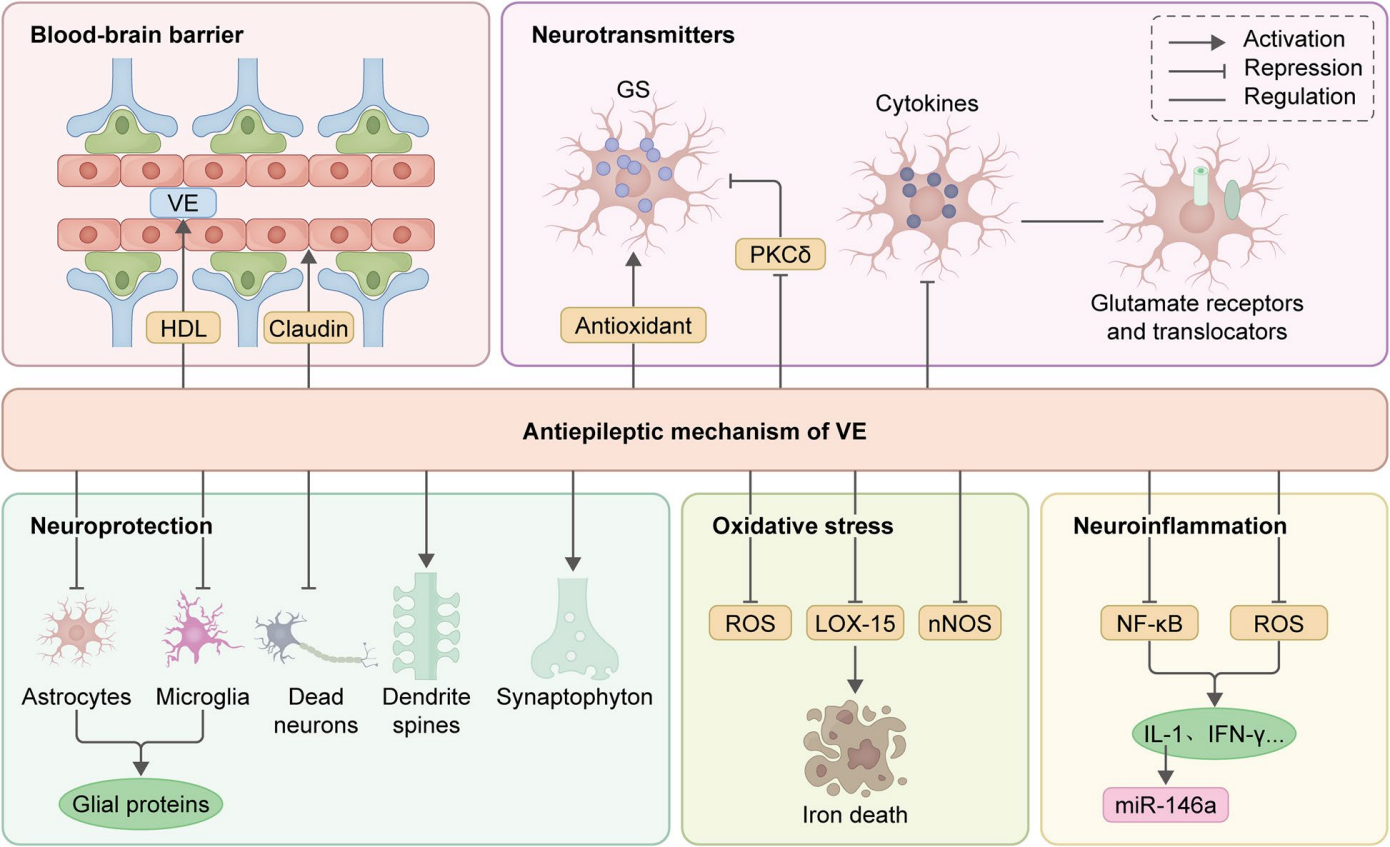
Schematic diagram depicting the antiepileptic mechanism of VE.

VE has emerged as a compelling adjunctive therapeutic candidate in epilepsy, demonstrating modulatory potential across multiple epileptogenic pathways [112]. While mechanistic studies suggest that VE’s antioxidant properties attenuate lipid peroxidation cascades and mitochondrial dysfunction, emerging evidence positions VE as a pleiotropic regulator, suppressing glutamate excitotoxicity, stabilizing BBB integrity, and tempering IL-1β-mediated neuroinflammation (Fig. 8).

The antiepileptic mechanisms of VE could be categorized as protection of BBB integrity (VE crosses the BBB through high-density lipoprotein [HDL]-mediated mechanisms and could reverse the down-regulation of the BBB protein claudin), attenuating excitotoxicity (on the one hand, VE directly maintains the stability of GS content in astrocytes through antioxidant action; on the other hand, because the expression of PKCδ could reduce the expression of GS in astrocytes, VE could maintain neurotransmitter homeostasis by inhibiting PKCδ. VE also counteracts the ability of activated microglia to regulate glutamate receptors and transporter proteins of astrocytes via cytokines to influence neuronal excitability), neuroprotection (reducing the activation of astrocytes and microglia, decreasing the expression of neuroglial proteins; decreasing neuronal death; preventing loss of dendritic spines; and enhancing synaptophysin immunoreactivity), reducing OS (decreasing the levels of ROS and nNOS; and decreasing the levels of LOX-15 thereby preventing iron death) and alleviating neuroinflammation (inhibit secretion of various inflammatory mediators such as IL-1 and interferon-γ [IFN-γ] by inhibiting NF-κB activation and blocking ROS signaling; α-TOH could inhibit the up-regulation of miR-146a by decreasing the level of pro-inflammatory cytokines such as IL-1β).

#### VE exerts an antiepileptic effect by inhibiting OS

VE, particularly in the form of α-TOH, is recognized for its potent antioxidant properties, which play a crucial role in mitigating OS. Indeed, pretreatment with VE has been demonstrated in various experimental models to significantly reduce the generation of oxygen and nitrogen free radicals induced by seizures, with effects observable within a time frame of minutes to hours [53, 113]. Epileptic seizures in the rat model of kainic acid-induced epilepsy have been shown to lead to reductions in the hippocampus of the most abundant phospholipid species that contain polyunsaturated fatty acyl chains [114]. As a fat-soluble vitamin, VE is incorporated into cell membranes, where it protects polyunsaturated fatty acids from oxidative damage by scavenging free radicals and breaking the chain reaction of lipid peroxidation, which is critical for synaptic integrity [115]. In a study the treatment with α-TOH that lasted fifteen days post-ictal, significantly decreased the levels of protein carbonylation in hippocampi of epileptic rats, which is a robust oxidant stress hallmark [5]. Notably, this chain-breaking activity is amplified by synergistic interactions with ascorbic acid, which regenerates α-TOH from its oxidized tocopheryl radical, enhancing redox cycling efficacy [116].

Beyond direct radical neutralization, α-TOH also plays a significant part in suppressing ROS generation by inhibiting NADPH-OX isoforms, particularly NADPH-OX2 and NADPH-OX4, which are up-regulated during seizure-induced glutamate excitotoxicity [49]. NMDA receptor activation elevates intracellular Ca^2+^, triggering NADPH-OX-mediated O₂⁻ production and mitochondrial electron leakage. α-TOH curtails this cascade, reducing mitochondrial permeability transition pore (mPTP) opening and cytochrome c release, thereby attenuating apoptotic signaling [3]. Notably, the oxidized metabolite of α-TOH, α-tocopheryl quinone, demonstrates enhanced activity of inhibiting the 15-LOX mediated lipid signaling—a pathway implicated in neuronal death [11].

However, emerging evidence suggests that tocotrienols may possess even greater antioxidant potential due to their unique structure, which allows them to interact with lipid membranes more effectively and modulate inflammatory pathways [117]. The reducing abilities of tocotrienol isomers decrease in the order of α > β > γ > δ [111].

#### VE exerts antiepileptic effects by attenuating excitotoxicity

α-TOH demonstrates multimodal antiepileptic efficacy, which is partially mediated through astrocytic GS modulation within the glutamine-glutamate-GABA cycle [3]. Reduced GS activity compromises astrocytic homeostatic capacity, permitting extracellular glutamate accumulation and impairing neurotransmitter recycling, a dual pathology exacerbating both excitotoxicity and inhibitory network instability [42]. Preclinical evidence suggests that α-TOH administration attenuates this cascade by targeting protein kinase (PKCδ), a negative regulator of GS expression [118]. By inhibiting PKCδ overactivation in epileptogenic foci, α-TOH indirectly restores GS-mediated glutamate clearance. Additionally, VE attenuates excitotoxicity by inhibiting microglial activation, indirectly stabilizing glutamate transporters and receptor dynamics in astrocytes [3]. What’s more, one pivotal study utilized an epilepsy model in rats, where the administration of VE was found to modulate the levels of various trace elements in brain tissues. Results indicated that compared to the control and epilepsy groups, rats treated with VE exhibited significantly higher Mg^2+^ levels in their brain tissues [115], which is important in neuronal function and protection against excitotoxicity. In neurons, Mg^2+^ could attenuate glutamate excitotoxicity in cultured rat hippocampal neurons by blocking NMDA receptors and regulating mTOR signaling [45]. Additionally, a another study examined the role of VE in reducing ferroptosis—a form of regulated cell death associated with glutamate excitotoxicity—and found that VE treatment in pentylenetetrazole-kindled rats reduced seizure frequency and severity [11].

On the other hand, it can be hypothesized that α-TOH may influence the expression and/or activity of GABA and/or AMPA receptors. However, experimental evidence does not support direct α-TOH modulation of these receptors. In hippocampal epileptic tissues, α-TOH fails to attenuate GABA receptor desensitization or alter AMPA/GABA current ratios, despite marked excitatory-inhibitory imbalance. Electrophysiological analyses reveal no α-TOH-induced changes in GABA_A_ receptor subunit composition, phosphorylation states, or chloride ion homeostasis [5].

#### VE exerts antiepileptic effects by modulating inflammatory responses

Emerging evidence underscores α-TOH, the primary bioactive form of VE, as a potent modulator of neuroinflammatory cascades in TLE [5]. While its antioxidant properties are well characterized, α-TOH exerts antiepileptic effects through diverse anti-inflammatory mechanisms.

Ambrogini et al. demonstrated that α-TOH significantly attenuates hippocampal neuroinflammation by inhibiting astrogliosis and microglial reactivity [5]. SE reduces the expression of pro-inflammatory cytokines IL-1β and TNF-α, likely via indirect modulation of inflammatory signaling. Paradoxically, α-TOH up-regulates IL-6, a cytokine with dual pro- and anti-inflammatory effects [119]. While IL-6 may counteract neuroinflammation by down-regulating IL-1β/TNF-α through feedback loops or enhancing adenosine A1 receptor-mediated anticonvulsant effects [5, 120], its temporal dynamics in epileptogenesis warrant further investigation.

Intriguingly, α-TOH could modulate neuroinflammatory pathways via the regulation of microRNAs [5]. Notably, α-TOH suppresses miR-146a, a key mediator of cytokine signaling that is up-regulated during epileptogenesis [121]. This phenomenon is tentatively linked to its IL-1β-lowering effects rather than direct miRNA interactions. In addition, miR-146a inhibition subsequently increases IL-6 expression, creating a regulatory loop that down-regulates IL-1β/TNF-α production [5]. Moreover, α-TOH down-regulates miR-124 and miR-126, microRNAs implicated in neurovascular integrity and inflammatory responses. Interestingly, miR-124 plays dual and opposing roles in epileptogenesis—attenuating neuronal hyperexcitability yet exacerbating inflammation.

In addition, VE’s mechanism in combating inflammation partly lies in its potent antioxidant properties. By scavenging free radicals and ROS, VE mitigates OS, which is a significant contributor to inflammatory responses. Studies have shown that VE supplementation can decrease markers of OS, such as MDA, while increasing levels of endogenous antioxidants like glutathione [122]. This antioxidant activity not only protects cellular structures from damage but also modulates signaling pathways associated with inflammation.

#### VE exerts antiepileptic effects by ensuring the integrity of the BBB

OS is a critical factor contributing to the disruption of the BBB, leading to increased permeability and neuronal damage [123]. VE, as a fat-soluble antioxidant, can significantly lower levels of ROS and lipid peroxidation products in the brain, thereby preserving the structural integrity of the BBB [124].

In addition to its antioxidant properties, VE modulates various cellular signaling pathways that are crucial for maintaining BBB integrity. The phosphoinositide 3-kinase (PI3K)/Akt signaling pathway, which is known to regulate cell survival, growth, and metabolism, is one such pathway influenced by VE. Activation of the PI3K/Akt pathway has been associated with enhanced tight junction integrity and reduced BBB permeability. Studies have shown that VE can activate this pathway, leading to increased expression of tight junction proteins such as occludin and claudin-5, which are essential for maintaining the structural integrity of the BBB [125].

Unlike other VE compounds, T3-13'COOH, a natural metabolite of VE, has been shown to specifically activate (pregnane X receptor) PXR without being influenced by the pharmacological inhibition of PPARγ activity [126]. This nuclear receptor plays a crucial role in the metabolism of xenobiotics and is believed to regulate the transcriptional processes that control the CYP450-mediated metabolism of VE, potentially through the regulation of the CYP4F2 and CYP3A4 isoenzymes [127]. The activation of PXR could also up-regulate the expression P-gp, which is a critical element of BBB [128].

#### VE exerts antiepileptic effects through neuroprotection

VE demonstrates multimodal neuroprotective efficacy in epilepsy, though its mechanistic hierarchy remains incompletely mapped. seizure may induce maladaptive neuroplasticity characterized by dendritic spine loss in hippocampal CA1 neurons—a structural correlate of synaptic dysfunction and cognitive dysfunction [129]. Post-SE, dendritic spines density remains reduced for days, reflecting impaired recovery of synaptic connectivity. α-TOH administration, initiated post-SE, restored the dendritic spine density to near-control levels, suggesting that it promotes the formation of new spine rather than preventing acute loss. Concurrently, α-TOH attenuates SE-induced reductions in synaptophysin immunoreactivity, a synaptic vesicle marker indicative of synapse integrity [43], suggesting roles in synaptogenesis and synapse preservation. In another study, treatment with α-TOH proved to be effective in reducing neuronal degeneration and neurofilament degradation occurring after SE, which is derived from the anti-inflammatory and antioxidant effects of α-TOH [130]. In addition, preclinical evidence suggests α-TOH administration mitigates astrocytic reactivity and microglial NLRP3 inflammasome activation, thereby attenuating neuroglial crosstalk-driven excitotoxic cascades.

### Antiepileptic effects of B vitamins

#### VB1

VB1, or thiamine, is vital for carbohydrate metabolism and the proper functioning of the nervous system [131]. It serves as a coenzyme in the conversion of carbohydrates into glucose, which is the primary energy source for the body's cells. VB1 is also involved in the synthesis of neurotransmitters, which are essential for communication between nerve cells. A recent study revealed that higher dietary intake of VB1 was inversely associated with the risk of epilepsy, particularly in vulnerable populations such as the elderly and low-income groups [131]. Another study demonstrated that VB1 significantly reduced levels of 8-OHdG, caspase-3, NO, and cGMP in the brain while protecting against pentylenetetrazole (PTZ)-induced neurotoxicity and apoptosis in SH-SY5Y cells [54]. These findings suggest that VB1 supplementation may serve as a potential adjunct therapy for epilepsy by modulating OS and the NO/cGMP pathway.

#### VB6

VB6, or pyridoxine, is a key player in amino acid metabolism and the synthesis of neurotransmitters such as serotonin and GABA. Deficiency in VB6 has been linked to various forms of epilepsy, particularly in infants and children. Studies have shown that VB6 supplementation can significantly reduce seizure frequency in patients with VB6-dependent epilepsy, a condition characterized by seizures that are responsive to pyridoxine [132]. In addition, pyridoxal phosphate (PLP), the bioactive form, modulates neuroinflammation by suppressing TLR4/TAK1-driven NF-κB/JNK activation and NLRP3 inflammasome priming—mechanisms that attenuate IL-1β-mediated hippocampal hyperexcitability [55].

#### VB9

As a central role in one-carbon metabolism, VB9 (folic acid) has been shown to have an antioxidant and a neuroprotective role, though therapeutic applications remain mechanistically contested. VB9 deficiency has been associated with increased levels of homocysteine, which can induce inflammatory responses and contribute to neurodegeneration [133]. Supplementation with VB9 has been shown to lower homocysteine levels and subsequently reduce inflammatory markers in the brain, providing a protective effect against neuroinflammation [134]. On the other hand, VB9 is involved in DNA synthesis and repair, which is crucial for neuronal growth and function.

#### VB12

In human metabolism, VB12 (cobalamin) plays a crucial role as a cofactor in the conversion of methylmalonyl-CoA to succinyl-CoA, which is essential for energy production [135]. Additionally, VB12 works closely with folate in various metabolic processes, such as transsulfuration and transmethylation reactions, which are vital for maintaining healthy cellular function and proper amino acid metabolism [62]. These combined actions highlight the importance of both nutrients in supporting overall metabolic health. Beyond its classical function in myelin synthesis which is critical for preventing demyelination-associated hyperexcitability, VB12 deficiency disrupts homocysteine metabolism which is associated with an increased risk of neurological disorders, cardiovascular diseases, and cognitive decline [136]. Moreover, The low level of VB12 is associated with increased OS in chronic pancreatitis patients, which is manifested as a significant reduction in GSH, GPx and SOD [62].

### Antiepileptic effects of other vitamins

#### VC

VC, also known as AA, is essential for various physiological functions, including neurotransmitter synthesis, regulation of gene expression, and protection against oxidative stress. A wealth of research has indicated that VC may offer neuroprotective benefits in various excitotoxic neurological conditions. It has been suggested VC could prevent the occurrence of seizure activity induced by iron, methylmalonic acid, and pentylenetetrazol [137]. Similarly, in cases of cerebral ischemia, VC demonstrates protective effects against brain tissue injury [138]. However, in the model of penicillin-induced epileptiform activity, low-to-moderate doses (50–400 mg/kg) reduce the epileptiform activity frequency, optimal at 100 mg/kg. Moreover, lower (25 mg/kg) or higher doses (800 mg/kg) show no efficacy, emphasizing therapeutic window constraints [137].

As the most important water-soluble antioxidant in the brain extracellular fluid, VC can directly neutralize ROS and reactive nitrogen species (RNS), effectively reducing their harmful impact on cellular structures [139]. Furthermore, at synaptic interfaces, VC potentiates GABAergic inhibition via GABA_B1_ receptor up-regulation while competitively inhibiting NMDA-mediated calcium influx, a dual mechanism stabilizing excitatory-inhibitory equilibrium [8]. In addition, Emerging evidence positions VC as a neuroprotectant in postictal recovery, as it suppresses pro-apoptotic Bax/caspase-3 cascades while enhancing Bcl-2-mediated mitochondrial resilience [140].

#### VA

VA, a fat-soluble vitamin, is essential for various physiological processes, particularly in the CNS. Its active metabolite, retinoic acid, plays a crucial role in gene expression, influencing neuronal growth and differentiation. Research indicates that low levels of VA can impair the synthesis of retinoic acid, which is critical for normal brain function and development [141]. In animal models, VA deficiency has been shown to result in altered neuronal excitability and increased seizure frequency, underscoring the nutrient's protective role in the central nervous system [142]. The influence of VA on neural transmission is multifaceted, involving both direct and indirect pathways. For instance, VA has been shown to enhance the expression of genes involved in the synthesis of GABA, the primary inhibitory neurotransmitter in the brain. In Alzheimer's disease, the lack of VA would down-regulate the expression of GABA_Aα2_ and GABA_B1b_ receptors via retinoic acid-mediated signaling [9].

In addition to modulating glutamatergic-GABAergic equilibrium, VA supplement has been demonstrated to inhibit TLR4/MyD88/NF-κB pathway-mediated neuroinflammation via the activation of RARα [34]. Moreover, emerging data implicates gut-brain axis crosstalk, with VA deficiency altering fecal microbiota diversity (e.g. reduced Lactobacillus) and subsequent neuroinflammatory priming [9].

#### VK

VK, a fat-soluble vitamin, has traditionally been recognized for its role in coagulation [143]; however, its therapeutic potential in neurological disorders has garnered increasing attention in recent years. VK exists in two primary forms: phylloquinone (VK1) and menaquinone (VK2). Phylloquinone, predominantly found in green leafy vegetables, has a chemical structure characterized by a phytyl side chain, which is crucial for its biological activity. In contrast, menaquinone, which can be further classified into various subtypes (MK-4, MK-7, etc.), is synthesized by bacteria and is present in fermented foods and animal products [144].

VK1 and VK2 have emerged as a significant player in neuroprotection, particularly through its role in modulating neuroinflammatory pathways and OS. For instance, VK has been showed to suppress TLR2/TLR4-mediated NF-κB activation, attenuating hippocampal hyperexcitability while preserving Gas6/Axl/Akt pro-survival signaling,which is a dual mechanism mitigating both neuroinflammation and OS-induced neuronal apoptosis [12]. What’s more, recent research has uncovered that VK2 (MK-4) functions as an electron carrier in the mitochondrial electron transport chain, specifically bypassing complexes I and II via the Q cycle [145]. This unique mechanism helps address mitochondrial dysfunction by enhancing ATP production efficiency, effectively repairing cellular energy deficits. In contrast, MK-7, while also neuroprotective, appears to have a longer half-life and greater bioavailability, potentially leading to sustained effects over time. Studies suggest that MK-7 may enhance cognitive function by improving blood flow and supporting vascular health, which is crucial for maintaining neuronal integrity [146].

#### VQ

VQ, commonly known as coenzyme Q10 (CoQ10), is a fat-soluble vitamin-like compound that plays a crucial role in cellular energy production and antioxidant defense. In kainate-induced TLE, CoQ10 pretreatment reduces seizure severity and inhibit hippocampal neuronal loss and aberrant mossy fiber sprouting [64]. The neuroprotective effect of CoQ10 has partly been attributed to its antioxidant properties and anti-apoptotic action through stabilizing mitochondrial permeability transition pores and preventing cytochrome c release [147] CoQ10 can exist in three redox states: oxidized (ubiquinone), partially reduced (ubiquinol), and fully reduced (dihydroubiquinone). The interconversion between these states allows CoQ10 to effectively neutralize free radicals, thereby preventing lipid peroxidation and protecting cell membranes [148]. While antioxidant properties contribute significantly, CoQ10 has alse been found to modulate signaling pathways that are critical for neuronal survival. For example, it can influence the PI3K/Akt pathway, which is essential for cell survival and growth [149]. Activation of this pathway by CoQ10 can lead to increased neuronal resilience against various stressors, including excitotoxicity and inflammation. What’s more, CoQ10’s benefits may also arise from preserving glutamate transporter function, thereby curbing excitotoxicity [150].

## The potential effects of ASMs on the vitamin levels in the patients

### ASMs and VD

The association between chronic antiseizure medication use and hypovitaminosis D in epilepsy populations has been established epidemiologically, although mechanistic interpretations remain debated. A 2016 Malaysian cohort study of 244 pediatric epilepsy patients (aged 3–18 years) receiving ≥ 1 year of ASM therapy revealed that 22.5% exhibited VD deficiency (25[OH]D levels ≤ 35 nmol/L) with an additional 19.7% demonstrating insufficiency (25[OH]D levels of 36–50 nmol/L), establishing vulnerability in this population [151]. Notably, the cross-sectional design and single-center recruitment limit generalizability, while seasonal variation in subtropical regions may confound baseline VD status—methodological constraints requiring consideration in comparative analyses. Notably, patients using enzyme inducing ASMs (EIASMs) have a greater risk of VD deficiency than those using Non-EIASMs [152].

The pharmacokinetic interplay between ASMs and VD metabolism involves multi-organ enzymatic regulation. Hepatic 25-hydroxylation via cytochrome P450 isoform CYP2R1 converts cholecalciferol to 25(OH)D, followed by renal 1α-hydroxylation through CYP27B1 to yield bioactive 1,25(OH)2D [153]. Paradoxically, the VD catabolic pathway mediated by CYP24A1—a mitochondrial enzyme responsible for 24-hydroxylation of both 25(OH)D and 1,25(OH)2D—appears to be synergistically up-regulated by classical EIASMs [152]. Experimental models have demonstrated that phenytoin and carbamazepine activate the nuclear receptors PXR and constitutive androstane receptor (CAR), inducing CYP3A4-mediated VD hydroxylation and CYP24A1 overexpression through genomic and post-translational mechanisms [152]. This dual induction creates a metabolic sink, shunting VD toward inactive 24,25(OH)2D3 rather than the hormonal 1,25(OH)2D3.

### ASMs and VK

The interplay between VK and ASMs is a critical area of investigation, particularly given the potential for drug interactions that may influence treatment outcomes. Certain ASMs, particularly those that induce hepatic enzymes, can significantly alter the metabolism of VK, leading to deficiencies that may exacerbate seizure activity [154]. For instance, the use of enzyme-inducing ASMs such as phenytoin and carbamazepine has been associated with decreased levels of VK-dependent proteins, which are essential for maintaining neurological health and preventing seizures. Furthermore, the safety profile of combining VK with various ASMs has been a subject of concern. Research indicates that patients receiving VK antagonists and ASMs may face an increased risk of bleeding complications, particularly when VK levels are not adequately maintained [13].

## Conclusions

In this review, vitamins were shown to exhibit multifaceted antiepileptic properties, targeting interconnected pathways in seizure pathophysiology. VD and VE emerge as central players, demonstrating pleiotropic mechanisms. VD modulates neuroinflammation by suppressing TLR/NF-κB signaling, reducing pro-inflammatory cytokines (IL-1β and TNF-α) while simultaneously enhancing neurotrophin expression (BDNF and NT-3) and stabilizing calcium homeostasis through L-type channel regulation. VE, beyond its antioxidant role, mitigates excitotoxicity by preserving astrocytic GS activity and attenuating lipid peroxidation via PKCδ inhibition. Critically, VD and VE also reinforce BBB integrity by up-regulating tight junction proteins (claudin-5, ZO-1) and suppressing MMP-9, disrupting the albumin-TGFβ-driven hyperexcitability cycle. B vitamins contribute uniquely: VB6 suppresses TLR4/TAK1-driven NF-κB/JNK activation and NLRP3 inflammasome priming, VB1 modulates OS and the NO/cGMP pathway, and VB9/12 modulate homocysteine metabolism, commonly reducing neuroinflammatory cascades. VC and VQ counteract OS by scavenging ROS and enhancing endogenous antioxidants (SOD, glutathione), while VA promotes GABAergic neuron differentiation. Emerging evidence further implicates gut microbiota modulation as a therapeutic axis, with VD and B vitamins influencing microbial diversity (e.g. Bacteroidetes reduction, Bifidobacterium enrichment), potentially altering seizure susceptibility.

Chronic ASM use profoundly disrupts vitamin metabolism, creating a self-perpetuating cycle of deficiency and disease progression. VD is particularly vulnerable: enzyme-inducing ASMs (e.g., phenytoin and carbamazepine) activate PXR pathways, which up-regulate CYP24A1 and CYP3A4. This accelerates VD catabolism to inactive 24,25(OH)₂D₃, exacerbating deficiency, as evidenced in a Malaysian cohort in which 42% of pediatric epilepsy patients had suboptimal VD levels. VK dynamics are similarly perturbed. Hepatic enzyme-inducing ASMs reduce levels of VK-dependent proteins, critical for vascular and neuronal health, while increasing bleeding risk in patients receiving VK antagonists. Less characterized are ASM effects on other vitamins. These interactions underscore the necessity of routine vitamin status monitoring in epilepsy management, particularly in resource-limited settings where deficiencies are prevalent.

Furthermore, it is crucial to evaluate the risks associated with vitamin supplementation, as excessive dosages can lead to adverse effects, including neuropathy or metabolic imbalances. For instance, maternal supplementation with high doses of α-TOH has been shown to impact cell signaling and synaptic plasticity in the developing hippocampus, leading to lasting negative effects in adult offspring [155]. Similarly, another study demonstrated that adult offspring born to mothers who received high doses of α-TOH during pregnancy and breastfeeding show lasting changes in the hippocampus, including an altered density of axo-spinous synapses and modified structural relationships between neurons and glial cells at synaptic sites [156]. These structural changes are associated with impaired long-term potentiation induction and deficits in spatial memory performance. Thus a comprehensive risk assessment should be conducted prior to initiating supplementation, especially in patients with comorbid conditions or those on multiple medications, to mitigate potential interactions and ensure safe management of their epilepsy.

The clinical application of vitamin supplementation in epilepsy requires a nuanced understanding of the specific types of epilepsy that may benefit from such interventions, particularly those classified as vitamin-dependent epilepsy, such as pyridoxine-dependent epilepsy [157]. Recommendations for vitamin supplementation should be tailored based on factors such as the patient's age, existing medical conditions, and concurrent medications. For instance, VD supplementation can vary significantly, with studies suggesting dosages ranging from 400 IU to 4000 IU daily, depending on the severity of deficiency and patient response [158]. Similarly, therapeutic VB6 dosages for patients with pyridoxine-dependent epilepsy typically range from 15 to 30 mg/kg/day in infants, with neonates receiving up to 200 mg/day and adults requiring doses as high as 500 mg/day [159]. However, the response to VB6 therapy can vary among patients with different genetic backgrounds, highlighting the need for personalized treatment plans. For example, patients with ALDH7A1 mutations may require additional dietary interventions, such as lysine restriction and arginine supplementation, to enhance seizure control and mitigate the effects of toxic metabolite accumulation [160].

Despite promising observations and foundational research supporting the role of vitamins in epilepsy management, there remains a notable gap in high-quality randomized controlled trials (RCTs) that can definitively establish the therapeutic benefits and optimal supplementation strategies. The current body of literature is predominantly composed of observational studies and basic research, which, while informative, do not provide the robust evidence required to shift clinical practice guidelines. Therefore, the call for more rigorous RCTs is imperative to substantiate the role of vitamins in epilepsy treatment and to refine the criteria for their clinical application.

This review provides a new direction for academic research and clinical practice of vitamins. It is essential to recognize that while the preliminary findings are promising, the current body of evidence remains in its infancy. The diverse mechanisms by which vitamins operate suggest a complex interplay that must be unraveled through rigorous scientific inquiry. Given the potential integration of vitamins into epilepsy treatment protocols, it is crucial to approach this development with a balanced perspective. On one hand, the promise of vitamin supplementation as adjunctive therapy could offer a low-risk, cost-effective strategy for improving outcomes in patients whose seizures are refractory to conventional treatments. On the other hand, the scientific community must exercise caution, ensuring that any recommendations are grounded in robust evidence derived from well-designed, large-scale clinical trials.

In conclusion, while the current evidence supports the potential role of vitamins in epilepsy management, it is imperative that the research community moves forward with a commitment to rigorous scientific inquiry and balanced interpretation of findings. By fostering a collaborative approach that integrates insights from basic science, clinical research, and patient-centered care, we may ultimately be able to elucidate the true potential of vitamins as part of a comprehensive epilepsy treatment strategy. This endeavor not only has potential as a new therapeutic options for patients but also has enriched our understanding of the underlying mechanisms of epilepsy, advancing the field as a whole.

## Data Availability

Availability of data and materials is not applicable in this study.

## Abbreviations

AA: Ascorbic acid
AJs: Adherens junctions
Alb: Albumin
AMPA receptor: α-Amino-3-hydroxy-5-methyl-4-isoazole receptor
AP-1: Activator protein-1
AQP4: Water channel protein-4
ASMs: Antiseizure medications
Bax: Bcl2-associated X
BBB: Blood-brain barrier
Bcl-2: B-cell lymphoma-2
BDNF: Brain-derived neurotrophic factor
CAT: Catalase
CBP: Calcium-binding proteins
cGMP: Cyclic guanosine monophosphate
CNTF: Ciliary neurotrophic factor
COX: Cyclooxygenase
DRE: Drug-resistant epilepsy
ECs: Endothelial cells
FMT: Fecal microbiota transplantation
GABA: γ-Aminobutyric acid
GAD1: Glutamic acid decarboxylase
GAT: GABA transporter
GCLC: Glutamate cysteine ligase
GDNF: Glial cell-derived neurotrophic factor
GFAP: Glial fiber acidic protein
GGT: γ-Glutamyl transpeptidase
Gln: Glutamine
Glu: Glutamate
GPx: Glutathioneperoxidase
GR: Glutathione reductase
GS: Glutamine synthetase
GSH: Glutathione
HDL: High-density lipoprotein
HO-1: Heme oxygenase-1
IFN-γ: Interferon-γ
IKK: IκB kinase
IL: Interleukin
iNOS: Inducible nitric oxide synthase
Kir 4.1: Inwardly rectifying potassium channel 4.1
LOX-15: Lipoxygenase 15
LPS: Lipopolysaccharides
L-VSCC: L-type voltage-sensitive Ca^2+^ channel
MAPK: Mitogen-activated protein kinase
MDA: Malondialdehyde
MFS: Moss fiber germination
MMP: Matrix metalloproteinase
mPTP: Mitochondrial permeability transition pore
MyD88: Myeloid differentiation primary response gene 88
MADPH: Nicotinamide adenine dinucleotide phosphate
NADPH-OX: Nicotinamide adenine dinucleotide phosphoribonucleotide oxidase
NF-κB: Nuclear factor kappa-B
NGF: Nerve growth factor
NLR: Nucleotide-binding oligomerization domain-like receptors
NMDA receptor: N-methyl-D-aspartate receptor
NO: Nitric oxide
NSCs: Neural stem cells
nNOS: Neuronal-type nitric oxide synthase
NOS: Nitric oxide synthase
NR2 A/B: NMDA receptor subunits 2A and 2B
NSCs: Neural stem cells
NT: Neurotrophin
OL: Oligodendrocyte
OPN: Osteopontin
OS: Oxidative stress
PARP-1: Poly ADP-ribose polymerase-1
P-gp: P glycoprotein
PLP: Pyridoxal phosphate
PKCδ: Protein kinase Cδ
PRRs: Pattern recognition receptors
RARα: Retinoic acid receptor α
RIP-1: Receptor-interacting protein 1
RIPK2: Receptor-interacting serine/threonine-protein kinase 2
RNS: Reactive nitrogen species
ROS: Reactive oxygen species
SCFAs: Short-chain fatty acids
SIRT1: Silent mating type information regulation 2 homolog-1
SOD: Superoxide dismutase
TAB: TAK-binding proteins
TAK1: Transforming growth factor-β-activated kinase 1
THEM4: Thioredoxin superfamily member 4
TIRAP: Toll interleukin 1 receptor domain containing adapter protein
TJs: Tight junctions
TLE: Temporal lobe epilepsy
TLR: Toll-like receptor
TNF-α: Tumor necrosis factor-α
TRAF6: Tumor necrosis factor receptor-associated factor 6
VD: Vitamin D
VDH: Vitamin D hormone
VDR: Vitamin D receptor
VDREs: Vitamin D response elements
ZO: Zonula occludens
